# Inhibition of SIRT7 Overcomes Radioresistance in Pancreatic Neuroendocrine Tumors by Reactivating MEN1 Expression

**DOI:** 10.1002/advs.202519824

**Published:** 2026-04-13

**Authors:** Jianyun Jiang, Yan Wang, Yi Qin, Luohai Chen, Xiaowu Xu, Guixiong Fan, Desheng Jing, Miaoyan Wei, Xianjun Yu, Junfeng Xu, Shunrong Ji, Jie Chen

**Affiliations:** ^1^ Center For Neuroendocrine Tumors Fudan University Shanghai Cancer Center Shanghai China; ^2^ Department of Oncology, Shanghai Medical College Fudan University Shanghai China; ^3^ Department of Pancreatic Surgery Fudan University Shanghai Cancer Center Shanghai China; ^4^ Shanghai Pancreatic Cancer Institute Shanghai China; ^5^ Pancreatic Cancer Institute Fudan University Shanghai China

**Keywords:** DNA damage response, MEN1, pancreatic neuroendocrine tumors, radiotherapy, SIRT7

## Abstract

Pancreatic neuroendocrine tumors (PanNETs) frequently exhibit loss or reduced expression of the tumor suppressor MEN1, a key regulator of tumor progression and DNA damage response (DDR). However, the upstream mechanisms driving MEN1 silencing remain unclear. Here, a pooled epigenetic CRISPR–Cas9 screen identified the NAD^+^‐dependent deacetylase SIRT7 as a critical suppressor of MEN1 expression. Mechanistically, SIRT7 interacts with DNMT1 and promotes catalytic activity‐dependent recruitment of DNMT1 to the MEN1 promoter, leading to promoter hypermethylation and transcriptional repression. Clinically, SIRT7 is overexpressed in PanNET tissues and inversely correlates with MEN1 levels and patient prognosis. Genetic or pharmacologic inhibition of SIRT7 restored MEN1 expression, reduced MRN complex abundance, and impaired double‐strand break repair. Graded MEN1 re‐expression demonstrated a quantitative relationship between MRN levels, DNA repair efficiency, and radiosensitivity. Functionally, SIRT7 inhibition enhanced radiation‐induced DNA damage and apoptosis in a partially MEN1‐dependent manner and significantly suppressed tumor growth in patient‐derived organoids and multiple independent xenograft models. Collectively, these findings define a catalytic activity‐dependent SIRT7–DNMT1–MEN1 epigenetic axis that modulates DDR and radiosensitivity, supporting SIRT7 targeting as a strategy to improve radiotherapy efficacy in PanNETs.

## Graphical abstract

Pancreatic neuroendocrine tumors frequently silence MEN1 through epigenetic mechanisms. Here, SIRT7 recruits DNMT1 to the MEN1 promoter, drives hypermethylation, and enhances DNA repair. Inhibiting SIRT7 restores MEN1, reduces MRN complex abundance, impairs double‐strand break repair, and sensitizes PanNET models to radiation, supporting SIRT7 as a promising radiosensitizing target.

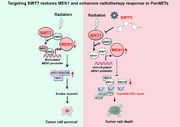

## 1 Introduction

Pancreatic neuroendocrine tumors (PanNETs) are biologically heterogeneous neoplasms whose clinical behavior is influenced by both genetic drivers and non‐genetic modifiers. Although considerable progress has been made in elucidating PanNET biology, treatment options—especially for advanced or metastatic disease—remain limited [1, 2]. Current management includes surgical resection and systemic therapies, yet radiotherapy has a limited and variably defined role in PanNET care and is less extensively discussed in major guidelines, reflecting the scarcity of prospective evidence and standardized radiation strategies for this disease [3, 4, 5, 6].

The MEN1 gene represents the most commonly mutated tumor suppressor in PanNETs, with somatic inactivation—through mutations or copy number variations—occurring in over one‐third of sporadic cases [7]. Loss of its protein product, menin, drives malignant progression and is associated with elevated metastatic potential and higher risk of recurrence [8, 9]. Beyond its established roles in transcriptional and signaling regulation (including TGF‐β and Hedgehog pathways), menin is also an essential component of the DNA damage response (DDR), a key determinant of cellular radiosensitivity [10, 11]. In our recent proteogenomic study of 108 nonfunctional PanNETs, we further demonstrated that MEN1 expression is altered in 32% of cases, with consequential proteomic changes impacting cellular stress response pathways [12]. Intriguingly, among a cohort of 169 sporadic PanNET patients, approximately 80% exhibited abnormal menin expression, while only about 30% harbored MEN1 gene mutations, suggesting a substantial role for epigenetic mechanisms in menin dysregulation [13]. This discrepancy highlights the critical need to elucidate the nongenetic pathways controlling MEN1 expression. Previous studies from our group revealed that the menin protein undergoes ubiquitin proteasome system (UPS) mediated degradation via the CUL4B–DCAF7 axis [14]. However, the mechanisms governing MEN1 transcriptional repression in PanNETs remain poorly characterized, limiting the development of targeted strategies for MEN1‐deficient tumors.

Emerging evidence indicates that DNA methylation at CpG islands in the MEN1 promoter region can suppress gene activation and lead to reduced MEN1 expression [15, 16, 17]. Nevertheless, the upstream regulators responsible for orchestrating this epigenetic silencing in PanNETs remain largely unidentified. To address this knowledge gap, we conducted a pooled epigenetic CRISPR‐Cas9 screen in BON‐1 cells to systematically identify modulators of MEN1 expression. Using FACS‐based sorting and deep sequencing, we identified SIRT7—a nuclear NAD^+^‐dependent deacetylase with recognized oncogenic roles in various cancers but poorly characterized functions in PanNETs [18]. Sirtuins, including SIRT7, are established regulators of cellular stress responses, such as DDR and endoplasmic reticulum stress (ERS), and have been linked to radioresistance in multiple malignancies [18, 19, 20, 21, 22, 23]. Recent reviews have further emphasized that sirtuins are central regulators of DNA repair, genome stability, transcriptional control, and stress adaptation, while also exhibiting context‐dependent tumor‐promoting or tumor‐suppressive functions in cancer [24, 25]. These studies also highlight the growing translational interest in isoform‐selective sirtuin modulators, including both inhibitors and activators, as emerging therapeutic tools in oncology and other complex diseases [25]. However, whether and how SIRT7 contributes to MEN1 dysregulation and DDR control in PanNETs has remained unclear.

In this study, we investigate the mechanistic basis and therapeutic implications of SIRT7‐mediated MEN1 regulation. Specifically, we examine how SIRT7 interfaces with DNMT1‐dependent promoter methylation to control MEN1 expression and DDR‐associated phenotypes, including radiosensitivity. Importantly, the relevance of SIRT7 to DDR extends beyond MEN1: prior work has shown that SIRT7 is recruited to DNA double‐strand breaks (DSBs) in a PARP1‐dependent manner and can promote chromatin remodeling and repair through deacetylation of H3K18ac, thereby facilitating 53BP1 recruitment [26]. SIRT7 has also been reported to modulate ATM signaling by promoting ATM dephosphorylation/inactivation, shifting repair toward error‐prone nonhomologous end joining (NHEJ) and enhancing survival after irradiation [27]. In addition, stress‐induced SIRT7 activity can engage broader adaptive programs, such as the IRE1α–XBP1 arm of the unfolded protein response, with downstream effects on immune evasion and apoptosis in other tumor contexts [28, 29]. Together, these studies support the concept that SIRT7 may influence DDR through multiple substrates, and therefore may regulate DDR in PanNETs through both MEN1‐dependent epigenetic control and MEN1‐independent mechanisms.

By defining how SIRT7–DNMT1–MEN1 signaling intersects with DNA repair capacity, this work addresses a critical knowledge gap with translational implications. Given the limited evidence base for radiotherapy in PanNETs and the need for effective radiosensitization strategies, targeting the SIRT7‐centered regulatory network may represent a rational approach to enhance treatment response. Here, we identify a previously unrecognized oncogenic role of SIRT7 in PanNETs and define its function as an epigenetic regulator of MEN1. We demonstrate that SIRT7 engages DNMT1 to establish promoter methylation–dependent repression of MEN1 in a catalytic activity–dependent manner, and we evaluate how disrupting this axis impacts DDR signaling and radiosensitivity in PanNET models. Collectively, these findings position the SIRT7–DNMT1–MEN1 pathway as a central epigenetic mechanism linking MEN1 suppression to dysregulated DNA damage response and therapeutic resistance in PanNETs.

## 2 Results

### 2.1 FACS‐based CRISPR‐Cas9 Screening Approach Identifies SIRT7 as a Key Suppressor of MEN1

**FIGURE 1:**
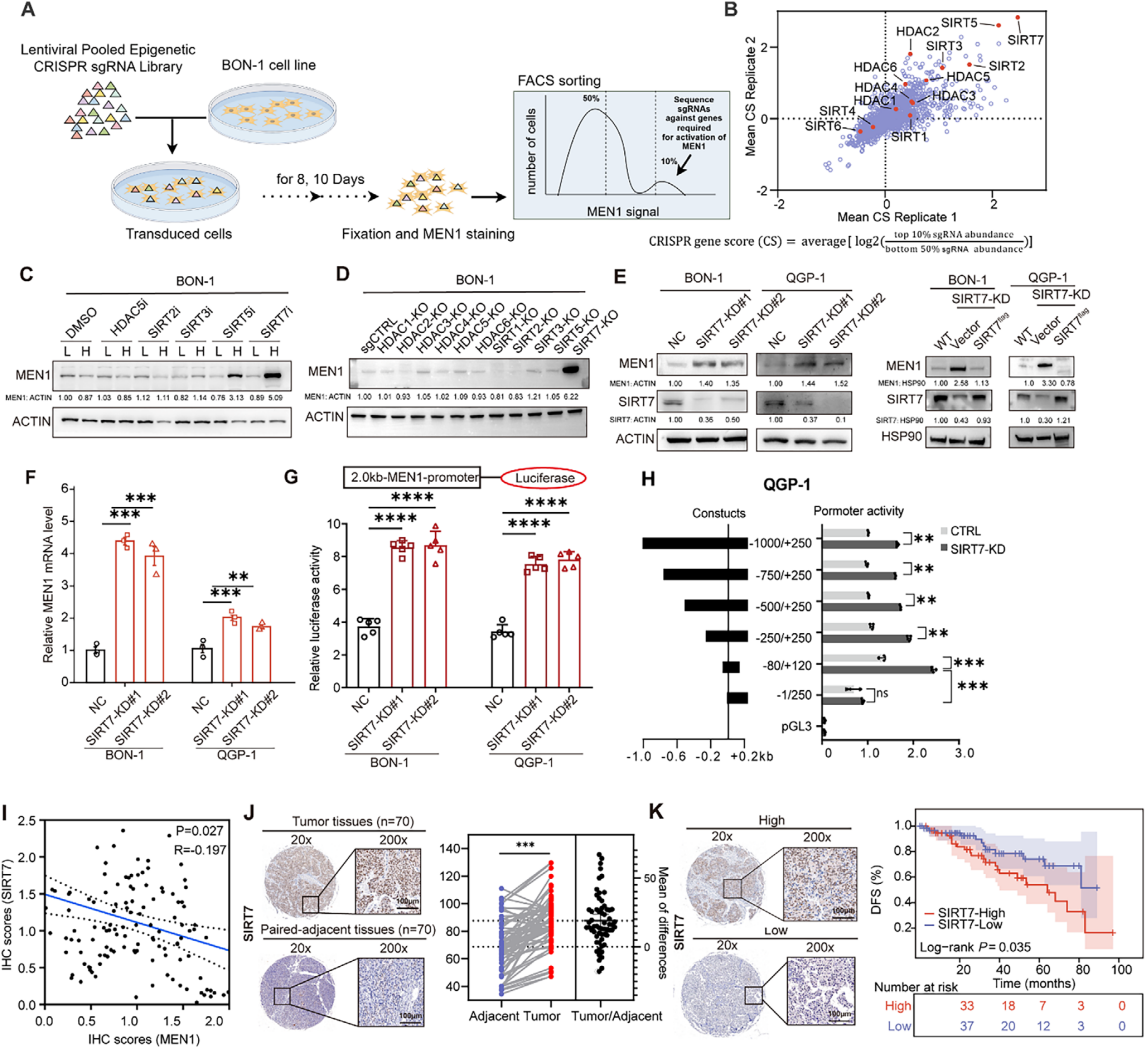
A FACS‐based CRISPR screen identifies SIRT7 as a transcriptional repressor of MEN1 and reveals its clinical significance in PanNETs. (A) Schematic of the FACS‐coupled pooled CRISPR–Cas9 screen in BON‐1 cells. (B) Scatter plot of CRISPR gene scores (CS) from two independent screens (Replicate 1 vs. Replicate 2); selected HDAC and sirtuin hits are indicated. (C) Representative immunoblot of MEN1 in BON‐1 cells treated with small‐molecule inhibitors targeting HDAC5, SIRT2, SIRT3, SIRT5, or SIRT7 at low (L) and high (H) doses. *n* = 3 independent experiments. (D) Immunoblot analysis of MEN1 protein in BON‐1 cells after individual CRISPR‐Cas9 knockout of selected HDACs and sirtuins. *n* = 3 independent experiments. (E) Immunoblot analysis of SIRT7 and MEN1 in BON‐1 and QGP‐1 cells after SIRT7 knockdown (SIRT7‐KD#1, SIRT7‐KD#2) and rescue by re‐expression of SIRT7 (SIRT7^Flag^) in SIRT7‐KD cells; Vector denotes empty‐vector rescue control. n = 3 independent experiments. (F) RT–qPCR analysis of MEN1 mRNA in BON‐1 and QGP‐1 cells after SIRT7 knockdown. n = 3 biologically independent samples per group. Statistics: one‐way ANOVA. (G) Luciferase reporter assay measuring MEN1 promoter activity after SIRT7 knockdown. *n* = 5 biologically independent samples per group. Statistics: one‐way ANOVA. (H) MEN1 promoter truncation luciferase assays in QGP‐1 cells comparing CTRL versus SIRT7‐KD across indicated promoter fragments. *n* = 3 biologically independent samples per construct. Statistics: one‐way ANOVA. (I) Correlation between SIRT7 and MEN1 IHC scores in a PanNET tissue microarray (TMA; *n* = 121). Pearson correlation: *R* = −0.197, *p* = 0.027. (J) Representative IHC images of SIRT7 in paired tumor and adjacent tissues from a PanNET cohort (*n* = 70 pairs) with quantification. Images are shown at 20× and 200× magnification; scale bars are indicated in the images (200×, 100 µm). Statistics: two‐sided paired t‐test. (K) Kaplan–Meier analysis of disease‐free survival (DFS) stratified by SIRT7‐high vs SIRT7‐low expression in the PanNET TMA cohort (*n* = 121); log‐rank *p* = 0.035. Representative IHC images for SIRT7‐high and SIRT7‐low groups are shown at 20× and 200× magnification; scale bars are indicated in the images (200×, 100 µm). Statistics: log‐rank test. Data are presented as mean ± SEM unless otherwise indicated; ns, not significant; *p < 0.05, **p < 0.01, ***p < 0.001.

To identify epigenetic regulators of MEN1 gene expression, we employed a fluorescence‐activated cell sorting (FACS)‐based CRISPR–Cas9 screening approach [30], with MEN1 protein expression levels as the functional readout. The human PanNET cell line BON‐1 was transduced with a lentiviral pooled sgRNA library targeting 2508 epigenetic regulators (see Table S1 for a full list). To capture both immediate and delayed effects of these perturbations, cells were harvested at 8 and 10 days post‐transduction, allowing time for chromatin state changes while minimizing the loss of sgRNAs targeting essential regulators (Figure 1A, left panel). After fixation and immunostaining with a MEN1‐specific antibody, the cells were separated by flow cytometry into two fractions: the 50% lowest and 10% highest MEN1 expression levels (Figure 1A, right panel). The “CRISPR score” (CS) for each gene was calculated by determining the mean log_2_ fold change in sgRNA abundance between these fractions across all time points. Genes whose CRISPR knockout resulted in an increase in MEN1 expression (enriched sgRNAs in the high MEN1 expression fraction) were classified as negative regulators of MEN1 expression. Replicate screens were compared to assess reproducibility and analyzed using MAGeCK to identify statistically significant hits [31]. Notably, sgRNAs targeting HDAC and sirtuin family members were significantly enriched (Figure 1B), implicating these epigenetic regulators in MEN1 repression.

We next carried out orthogonal validation. siRNA knockdown of multiple hits (HDAC5, SIRT2, SIRT3, SIRT5, and SIRT7) increased MEN1 protein levels (Figure S1A). We then tested small‐molecule inhibitors of these targets at both low and high concentrations. Inhibition of SIRT5 and SIRT7 robustly increased MEN1, whereas SIRT2 and SIRT3 inhibition showed little effect (Figure 1C). Because genetic perturbations (CRISPR/siRNA) reflect sustained loss of function, while inhibitors act acutely and depend on intracellular potency and selectivity [32, 33], we further prioritized candidates using individual gene knockouts. We performed individual CRISPR–Cas9 knockout of SIRT2, SIRT3, SIRT5, and SIRT7. Only SIRT7 knockout consistently and strongly increased MEN1 expression (Figure 1D).

Given the inconsistent results for SIRT5 across orthogonal assays, we further tested whether SIRT5 truly regulates MEN1 transcription. In BON‐1 and QGP‐1 cells, two independent SIRT5 knockdown constructs (SIRT5‐KD#1 and SIRT5‐KD#2) did not increase MEN1 expression and did not alter MEN1 promoter luciferase activity (Figure S1B,C). Clinically, SIRT5 IHC showed no correlation with MEN1 levels in the PanNET tissue microarray cohort (*n* = 121; *R* = 0.048, *p* = 0.587; Figure S1D). In paired specimens, SIRT5 expression was not consistently different between tumor and adjacent tissues (*n* = 70 pairs; Figure S1E,F), and SIRT5 levels were not associated with patient prognosis in the full cohort (Figure S1G,H). These data do not support a major role for SIRT5 as an upstream regulator of MEN1 in PanNETs under the conditions tested, although a more subtle or context‐dependent effect cannot be completely excluded.

We therefore focused on SIRT7. We established two SIRT7‐knockdown cell lines (SIRT7‐KD#1 and KD#2). Western blot confirmed that SIRT7 knockdown upregulated MEN1 protein expression, while re‐overexpression of SIRT7 (SIRT7^Flag^) in the knockdown cells (SIRT7‐KD+SIRT7^Flag^) reduced MEN1 protein levels (Figure 1E). RT‐qPCR results were also consistent (Figures 1F and S1I,J). Similarly, MEN1 promoter luciferase reporter assays revealed that SIRT7 knockdown activated the promoter, whereas SIRT7 overexpression suppressed MEN1 transcription (Figures 1G and S1K). To localize the SIRT7‐responsive region, we performed serial deletion luciferase assays in QGP‐1 cells using promoter fragments spanning −1000/+250, −750/+250, −500/+250, −250/+250, −80/+120, and −1/+250 (with pGL3 as the control). SIRT7 knockdown increased promoter activity in all constructs retaining sequence upstream of −80 bp, whereas the −1/+250 fragment lost responsiveness (Figure 1H). Notably, the −80/+120 fragment remained fully responsive, indicating that the key SIRT7‐responsive element lies within the proximal promoter around the TSS.

Finally, we assessed clinical relevance in PanNET tissue microarrays (*n* = 121). SIRT7 protein levels inversely correlated with MEN1 (IHC score: *R* = −0.197, *p* = 0.027) (Figure 1I). We also observed that SIRT7 expression was higher in tumor tissues than in their paired normal tissues (*p* < 0.001) (Figure 1J). Patient clinicopathological characteristics were reported previously [34], and SIRT7 levels did not differ significantly across major clinicopathological categories (Table S2). In outcome analyses, high SIRT7 expression was associated with shorter disease‐free survival (DFS) (log‐rank test, *p* = 0.035; Figure 1K). After adjustment for age, sex, tumor location, and TNM staging variables, SIRT7‐high status remained an independent risk factor for recurrence (HR = 1.74, Table S3). These findings establish SIRT7 as a potent transcriptional suppressor of MEN1 expression and highlight its oncogenic function in PanNET pathogenesis.

### 2.2 SIRT7 Promotes Malignant Phenotypes in PanNETs In Vitro and In Vivo

**FIGURE 2:**
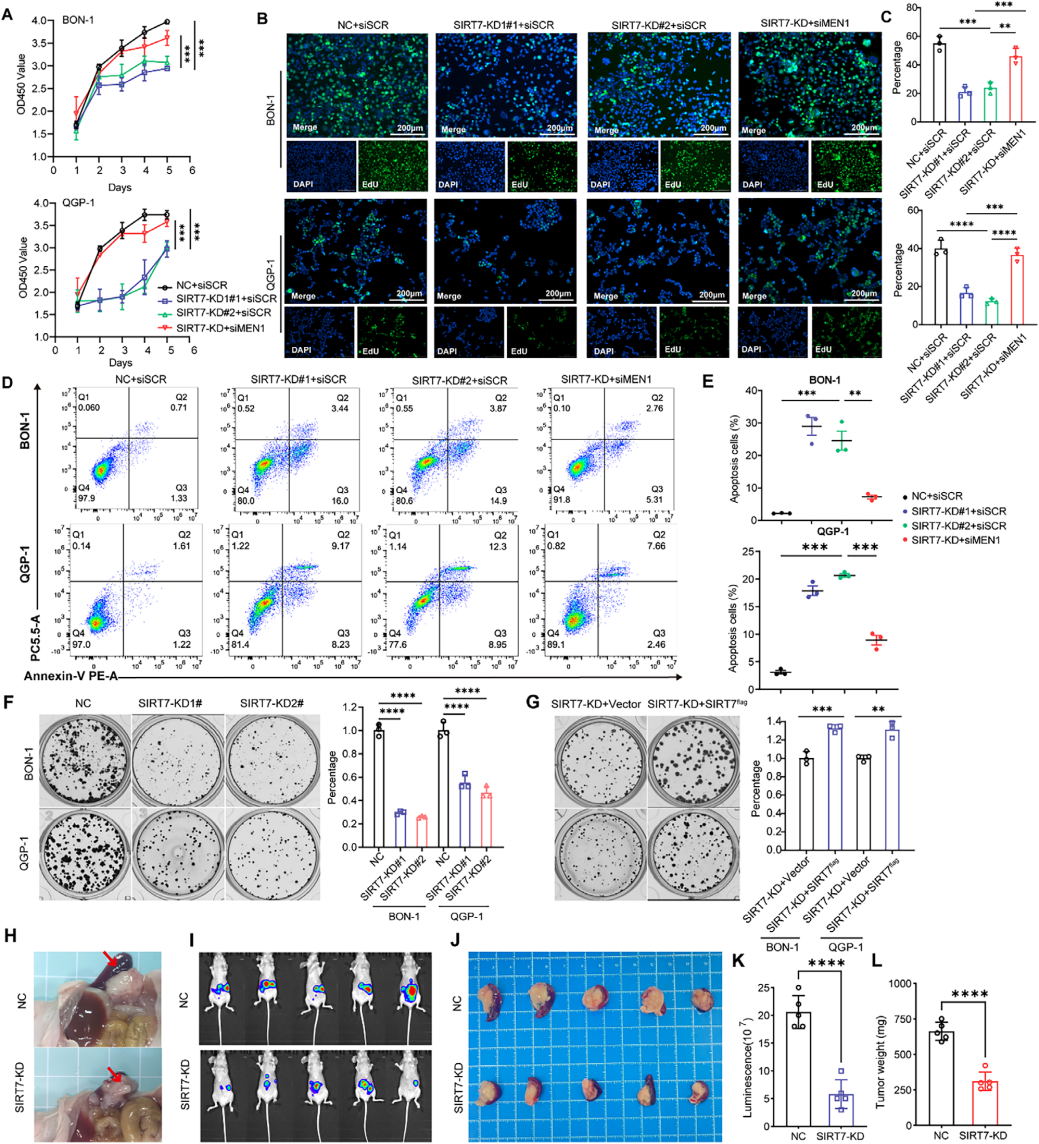
SIRT7 promotes malignant phenotypes in PanNETs in vitro and in vivo in a MEN1‐dependent manner. (A) Cell viability of BON‐1 and QGP‐1 cells measured by CCK‐8 under the indicated conditions. n = 3 biologically independent experiments. Statistics: one‐way ANOVA at each time point. (B) Representative images of EdU incorporation in BON‐1 and QGP‐1 cells under the indicated conditions. Images shown as DAPI, EdU, and merged channels. Scale bar, 200 µm. (C) Quantification of EdU‐positive cells corresponding to (B). Statistics: one‐way ANOVA. *n* = 3 biologically independent experiments. (D) Representative flow cytometry plots for Annexin V/PI apoptosis assays in BON‐1 and QGP‐1 cells under the indicated conditions. (E) Quantification of apoptotic cells corresponding to (D). Statistics: one‐way ANOVA. *n* = 3 biologically independent experiments. (F) Representative colony formation images of BON‐1 and QGP‐1 cells after SIRT7 knockdown with quantification shown at right. Statistics: one‐way ANOVA. *n* = 3 biologically independent experiments. (G) Colony formation rescue assay in SIRT7‐depleted cells re‐expressing SIRT7 (SIRT7^Flag^) or empty vector (Vector), with quantification shown at right. Statistics: one‐way ANOVA. *n* = 3 biologically independent experiments. (H) Representative gross anatomy images of orthotopic pancreatic tumors in mice (red arrows) generated from BON‐1 cells, comparing NC and SIRT7‐KD groups at endpoint (week 4). (I) Representative in vivo bioluminescence imaging of orthotopic tumors derived from BON‐1 cells at week 4 post‐implantation. (J) Representative macroscopic images of excised pancreatic tumors at week 4. (K) Quantification of bioluminescence intensity corresponding to (I). Statistics: unpaired two‐sided t‐test. *n* = 5 mice per group. (L) Endpoint tumor burden comparing NC and SIRT7‐KD groups. Statistics: unpaired two‐sided t‐test. *n* = 5 mice per group. Data are presented as mean ± SEM; ns, not significant; *p < 0.05, **p < 0.01, ***p < 0.001, ****p < 0.0001.

To investigate the oncogenic function of SIRT7 in PanNET cells and its interplay with MEN1, we performed a series of in vitro assays. SIRT7‐KD cells exhibited significantly reduced proliferation, as shown by decreased cell viability in CCK‐8 assays (Figure 2A) and lower DNA synthesis in EdU incorporation assays (Figures 2B,C). Western blot analysis confirmed that siMEN1 effectively reduced MEN1 protein levels in both control and SIRT7‐KD cells, verifying knockdown efficiency (Figure S2A). Importantly, siRNA‐mediated MEN1 knockdown in SIRT7‐KD cells rescued these proliferative defects (Figure 2A–C), supporting MEN1 as a major downstream mediator of SIRT7‐dependent growth phenotypes. Consistent with the reduced growth, flow cytometry indicated that SIRT7 knockdown increased the proportion of apoptotic cells, and MEN1 knockdown attenuated this effect (Figure 2D,E). Colony formation assays further revealed that SIRT7 knockdown markedly impaired clonogenic capacity in both PanNET cell lines (Figure 2F), whereas re‐expression of SIRT7 (SIRT7^Flag^) restored robust colony‐forming ability (Figure 2G). Additionally, pharmacological inhibition of SIRT7 using a small‐molecule inhibitor (compound 97491 at 10 µM for 48 h) produced the same effects as in genetic knockdown cells (Figure S2B–H), consistent with the oncogenic role of SIRT7.

We next evaluated the role of SIRT7 in an orthotopic xenograft model. Luciferase‐tagged control or SIRT7‐KD BON‐1 cells were implanted into the pancreata of nude mice, and tumor progression was monitored weekly via bioluminescence imaging (Figure 2H). By week 4, mice receiving SIRT7‐KD cells exhibited significantly lower bioluminescence signals than control mice (*n* = 5 mice per group, *p* < 0.05), indicating reduced orthotopic tumor growth (Figure 2I,K). Endpoint analysis showed that overall tumor burden was reduced in the SIRT7‐KD group compared to controls (Figure 2J,L). Collectively, these data indicate that SIRT7 drives malignant PanNET phenotypes both in vitro and in vivo.

### 2.3 SIRT7 Controls MEN1 Transcription by Recruiting DNMT1 to the MEN1 Promoter in PanNETs

**FIGURE 3:**
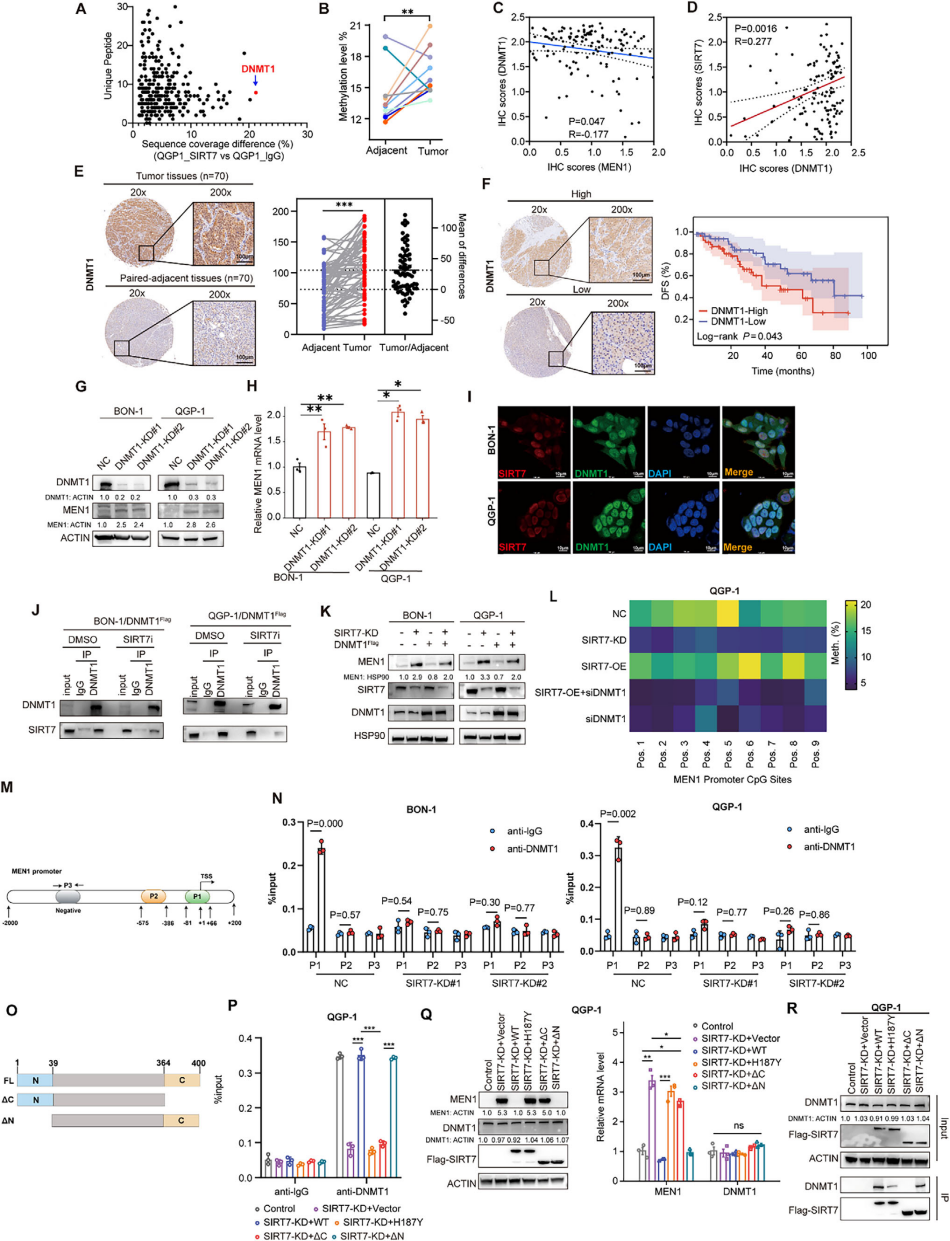
SIRT7 recruits DNMT1 to the MEN1 promoter to epigenetically silence MEN1 in PanNETs. (A) Immunoprecipitation–mass spectrometry (IP–MS) analysis in QGP‐1 cells identifies DNMT1 as a SIRT7‐interacting protein. Enrichment (sequence coverage difference) of proteins detected in anti‐SIRT7 IP versus IgG control in QGP‐1 cells; DNMT1 is highlighted. (B) Quantitative pyrosequencing of MEN1 promoter methylation in paired adjacent and tumor tissues from PanNET patients (*n* = 10 pairs). Statistics: two‐sided paired t‐test. (C,D) Correlation of IHC scores in PanNET TMA (n = 121): (C) DNMT1 versus MEN1 (Pearson *R* = −0.177, *p* = 0.047) and (D) DNMT1 versus SIRT7 (Pearson *R* = 0.277, *p* = 0.0016). (E) DNMT1 IHC in paired adjacent and tumor tissues (*n* = 70 pairs) with representative images and quantification. Representative images are shown at 20× and 200×; scale bar, 100 µm (200×). Statistics: two‐sided paired t‐test. (F) Kaplan–Meier analysis of disease‐free survival (DFS) stratified by DNMT1‐high versus DNMT1‐low expression in the PanNET TMA cohort (*n* = 121). Representative IHC images are shown at 20× and 200×; scale bar, 100 µm (200×). Statistics: log‐rank test, *p* = 0.043. (G) Immunoblot analysis of MEN1 in BON‐1 and QGP‐1 cells after stable DNMT1 knockdown (DNMT1‐KD#1, DNMT1‐KD#2) versus non‐targeting control (NC). n = 3 independent experiments. (H) RT–qPCR analysis of MEN1 mRNA levels in BON‐1 and QGP‐1 cells after DNMT1 knockdown. n = 3 biologically independent samples per group. Statistics: one‐way ANOVA. (I) Representative immunofluorescence images showing nuclear co‐localization of SIRT7 and DNMT1 in BON‐1 and QGP‐1 cells. Scale bar, 10 µm. n = 3 independent experiments. (J) Co‐immunoprecipitation (Co‐IP) assays validating SIRT7–DNMT1 interaction under the indicated conditions. n = 3 independent experiments. (K) DNMT1 overexpression (DNMT1^Flag^) reverses MEN1 upregulation caused by SIRT7 knockdown in BON‐1 and QGP‐1 cells. n = 3 independent experiments. (L) Heatmap of MEN1 promoter CpG methylation (Pos. 1–9) quantified by pyrosequencing in QGP‐1 cells under the indicated conditions. Values represent methylation percentage for each CpG position. *n* = 3 biologically independent samples per group. (M) Schematic of ChIP–qPCR primer locations at the MEN1 promoter: P1 (−81 bp), P2 (−575 bp), and a negative control region P3. (N) ChIP–qPCR analysis of DNMT1 occupancy at MEN1 promoter regions after SIRT7 knockdown. Data are shown as % input with IgG as control. *n* = 3 biologically independent samples per group. Statistics: unpaired two‐sided t‐test. (O) Schematic of SIRT7 constructs used for rescue: WT, catalytically inactive ΔC (aa 364–400 deletion) and ΔN (aa 1–39 deletion). (P) ChIP–qPCR analysis of DNMT1 occupancy at the MEN1 promoter in QGP‐1 cells across the indicated rescue conditions. *n* = 3 biologically independent samples per group. Statistics: one‐way ANOVA. (Q) Immunoblot and RT–qPCR analysis of MEN1 and DNMT1 in QGP‐1 cells across the rescue conditions. *n* = 3 independent experiments. (R) Co‐immunoprecipitation analysis of the interaction between DNMT1 and SIRT7 in QGP‐1 cells under the indicated rescue conditions. *n* = 3 independent experiments. Statistics: one‐way ANOVA. Data are presented as mean ± SEM unless otherwise indicated; ns, not significant; *p < 0.05, **p < 0.01, ***p < 0.001.

To investigate how SIRT7 silences MEN1, we performed immunoprecipitation–mass spectrometry (IP‐MS) in QGP‐1 cells stably expressing Flag‐tagged SIRT7 to identify SIRT7‐interacting proteins that may regulate MEN1 expression. We identified eight unique peptide sequences derived from DNMT1 (Figure 3A, Tables S5 and S6). DNMT1 is the principal maintenance methyltransferase in mammalian cells. Aberrant DNMT1 hyperactivation is a well‐established oncogenic driver that promotes epigenetic silencing of tumor suppressor genes through promoter hypermethylation [35]. Previous studies have shown that SIRT7 recruits heterochromatin‐associated complexes, including DNMT1, to rDNA regions to promote heterochromatin stabilization and epigenetic regulation [36, 37]. DNMT1 has also been implicated in MEN1 silencing in lung adenocarcinoma, where it binds directly to the MEN1 promoter and catalyzes CpG island hypermethylation [17]. However, whether a similar mechanism operates in PanNETs remains unclear.

To explore this possibility, we measured MEN1 promoter methylation in ten paired fresh‐frozen tumor and adjacent tissues using pyrosequencing. We initially profiled three pyrosequencing amplicons spanning the promoter–TSS region and observed the most consistent tumor–adjacent differences in the proximal promoter. We therefore focused subsequent CpG‐resolved analyses on the −67 to −2 region containing nine CpG sites. As shown in Figure 3B, tumor tissues exhibited significantly higher methylation than adjacent tissues (Table S7), and genomic coordinates and primer sequences are provided in Table S8. Reported methylation values represent the mean across the nine CpG sites. In addition, TMA‐based IHC analysis revealed a negative correlation between DNMT1 and MEN1 levels (Figure 3C) and a positive correlation between DNMT1 and SIRT7 levels (Figure 3D). DNMT1 expression was higher in tumor tissues than in corresponding adjacent non‐tumor tissues (Figure 3E), and elevated DNMT1 expression correlated with reduced DFS in PanNET patients (Figure 3F).

We generated DNMT1‐knockdown cells (DNMT1‐KD#1 and DNMT1‐KD#2) and measured MEN1 expression. DNMT1 depletion increased MEN1 protein levels compared with control cells (Figure 3G). RT‐qPCR analysis showed that MEN1 mRNA levels were also elevated after DNMT1 knockdown (Figure 3H, Figure S3A), supporting transcriptional regulation by DNMT1. Immunofluorescence staining revealed colocalization of SIRT7 and DNMT1 (Figure 3I, quantified in Table S9). Co‐immunoprecipitation assays further confirmed that SIRT7 physically interacts with DNMT1. Notably, in DNMT1^Flag^‐expressing BON‐1 and QGP‐1 cells, treatment with the SIRT7 inhibitor 97491 reduced the amount of SIRT7 co‐immunoprecipitated with DNMT1^Flag^ compared with DMSO controls (Figure 3J). Rescue experiments showed that DNMT1 overexpression reversed the increase in MEN1 expression caused by SIRT7 knockdown, supporting a model in which DNMT1 acts downstream of SIRT7 to regulate MEN1 expression (Figure 3K).

To establish a functional epigenetic link, we performed quantitative pyrosequencing of the MEN1 promoter in QGP‐1 cells, together with matched MEN1 expression measurements under five experimental conditions: control (NC), SIRT7‐knockdown (SIRT7‐KD), SIRT7 re‐expression (SIRT7‐OE: SIRT7‐KD + SIRT7^Flag^), SIRT7‐OE combined with siDNMT1, and siDNMT1 alone. SIRT7 knockdown significantly reduced MEN1 promoter methylation and increased MEN1 expression (Figure 3L, Figure S3B). Re‐expression of SIRT7 restored promoter methylation and suppressed MEN1 expression to baseline. Importantly, DNMT1 depletion in SIRT7‐OE cells prevented restoration of promoter methylation and maintained high MEN1 expression, indicating that SIRT7‐dependent methylation at the MEN1 promoter requires DNMT1. Pharmacological inhibition of SIRT7 with 97491 also decreased MEN1 promoter methylation in a dose‐dependent manner, further supporting that MEN1 promoter methylation is sensitive to SIRT7 enzymatic inhibition and is consistent with a requirement for SIRT7 catalytic activity (Figure S3C).

To examine region‐specific recruitment, we performed ChIP‐qPCR using two promoter regions (P1 and P2) and a negative control region (P3) (Figure 3M). All primer pairs (P1–P3) were validated on input DNA and produced single specific amplicons with comparable amplification efficiency (90%–110%). The P1 region (centered at −81 bp relative to the TSS) showed strong enrichment of DNMT1 in both cell lines, whereas P2 (−575 bp) and P3 showed minimal binding, indicating region‐specific co‐recruitment to the MEN1 promoter (Figure 3N). To further cross‐validate promoter methylation in an independent cohort, we analyzed a public PanNET dataset with matched Illumina 450K methylation and RNA expression data (GSE117853, *n* = 30). We identified nine CpG probes mapping within or adjacent to our pyrosequencing amplicon and stratified tumors by SIRT7 expression (top vs bottom tertile; *n* = 10 per group). Methylation was consistently higher in SIRT7‐high tumors across all nine probes (Δβ > 0 for 9/9 CpGs; median Δβ range 0.018–0.149), indicating a uniform hypermethylation trend in the same regulatory region (Figure S3D and Table S10). Although individual CpG associations did not remain significant after Benjamini–Hochberg correction (minimum FDR = 0.22), methylation showed a consistent direction across probes, supported by a directionality test (binomial *p* = 0.001953). In continuous correlation analyses, two CpG sites (cg00000289 and cg00000363) showed significant positive correlations with SIRT7 expression, whereas the remaining sites showed positive but non‐significant trends (Figure S3E).

Finally, we performed rescue experiments in SIRT7‐knockdown QGP‐1 cells using wild‐type SIRT7, a catalytically inactive mutant (H187Y), and truncation mutants lacking either the C‐terminal region (ΔC, aa 364–400) or the N‐terminal region (ΔN, aa 1–39) [38] (Figure 3O). SIRT7 knockdown markedly reduced DNMT1 occupancy at the MEN1 promoter (P1 region). Wild‐type SIRT7 restored DNMT1 enrichment, whereas the H187Y mutant failed to do so. Deletion of the C‐terminal region similarly impaired DNMT1 recruitment, indicating that both catalytic activity and an intact C‐terminal region of SIRT7 are required for efficient chromatin association (Figure 3P). Consistently, wild‐type SIRT7 restored repression of MEN1 at both mRNA and protein levels, whereas the H187Y and ΔC mutants failed to suppress MEN1 expression (Figure 3Q). Co‐immunoprecipitation assays (Figure 3R) further showed that DNMT1 binding was retained in WT SIRT7‐, H187Y‐, and ΔN‐reconstituted cells, but was markedly reduced in the ΔC mutant, supporting that the C‐terminal region of SIRT7 is required for efficient DNMT1 interaction. Collectively, these results support DNMT1 as an essential component of the SIRT7‐dependent epigenetic mechanism that represses MEN1 transcription in PanNET cells.

### 2.4 SIRT7 knockdown Downregulates DNA Damage Repair Genes in PanNET Cells

**FIGURE 4:**
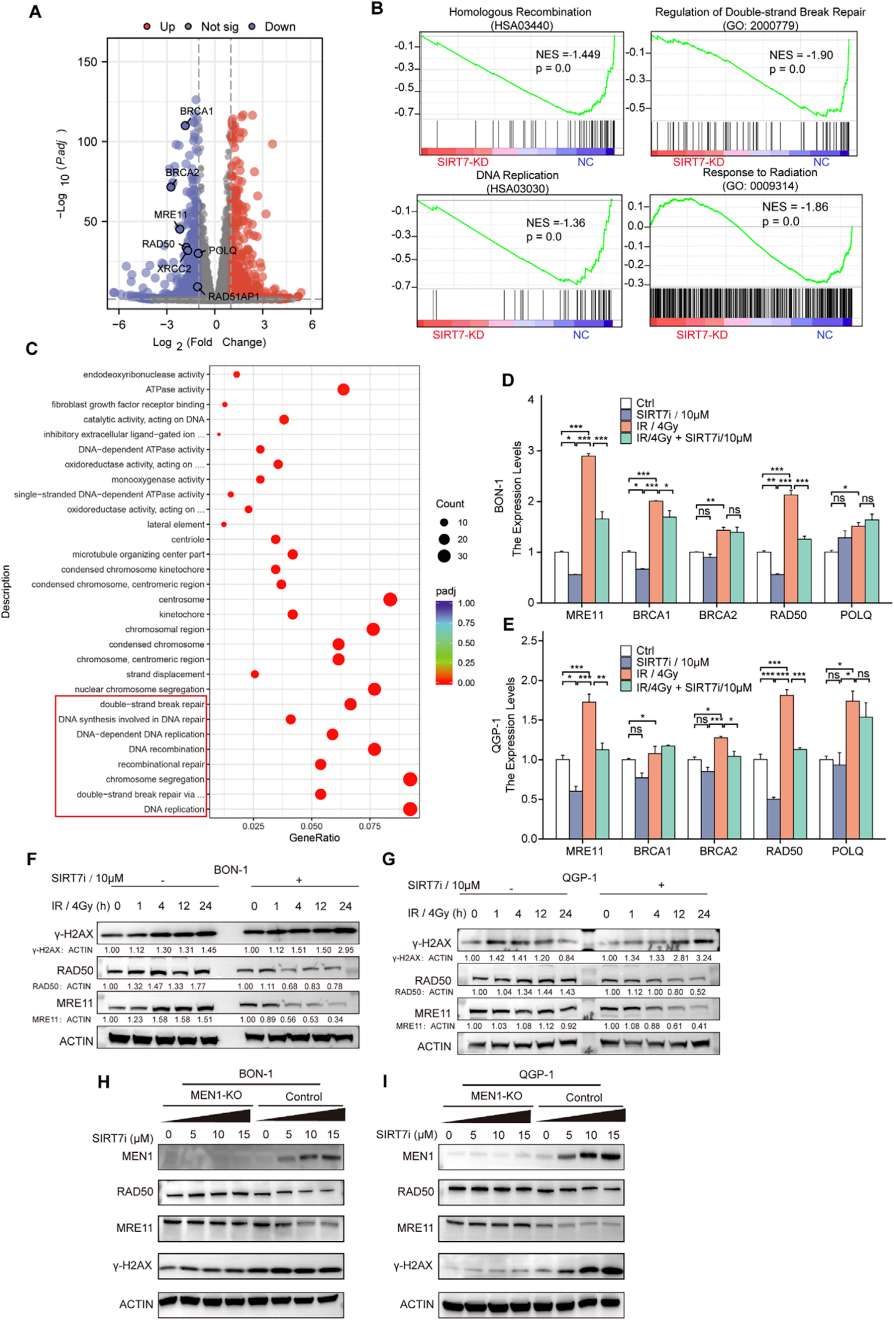
SIRT7 inhibition suppresses DNA damage repair pathways and sensitizes PanNET cells to irradiation. (A) Volcano plot from RNA‐seq comparing SIRT7‐knockdown (SIRT7‐KD) versus control (NC) in QGP‐1cells, highlighting representative DNA damage response (DDR) genes. *n* = 3 biologically independent samples per group. (B) Gene set enrichment analysis (GSEA) showing pathway‐level changes in SIRT7‐KD versus control cells. *n* = 3 biologically independent samples per group. (C) GO enrichment analysis of downregulated biological processes in SIRT7‐KD cells versus controls, with DDR‐related terms highlighted. *n* = 3 biologically independent samples per group. (D,E) RT–qPCR validation of DDR gene expression in BON‐1 (D) and QGP‐1 (E) cells treated with vehicle (Ctrl), SIRT7 inhibitor (SIRT7i, 10 µM), irradiation (IR, 4 Gy), or the combination (IR / 4 Gy + SIRT7i / 10 µM). Cells were collected 24 h after irradiation. *n* = 3 biologically independent experiments. Statistics: one‐way ANOVA. (F, G) Time‐course immunoblot analysis of γ‐H2AX, RAD50, and MRE11 in BON‐1 (F) and QGP‐1 (G) cells treated with IR (4 Gy) in the absence or presence of SIRT7i (10 µM) at the indicated times (0, 1, 4, 12, 24 h). *n* = 3 independent experiments. (H,I) Dose‐response immunoblot analysis in BON‐1 (H) and QGP‐1 (I) cells treated with increasing concentrations of SIRT7i (0, 5, 10, 15 µM) in Control (Cas9/vector) cells or MEN1‐knockout (MEN1‐KO) cells generated by CRISPR–Cas9. *n* = 3 independent experiments. Data are presented as mean ± SEM unless otherwise indicated. Significance is shown in the figure; ns, not significant; *p < 0.05, **p < 0.01, ***p < 0.001.

To assess how SIRT7 loss reshapes gene expression in PanNET cells, we performed RNA‐seq in SIRT7‐knockdown QGP‐1 cells and compared them with controls. In total, 1960 genes were significantly altered (1266 upregulated and 694 downregulated; |log2FC| > 1, FDR < 0.05). Notably, multiple DNA damage response (DDR) components were downregulated, including homologous recombination genes (BRCA1, BRCA2), MRN complex members (MRE11, RAD50), non‐homologous end joining factors (XRCC4), DNA single‐strand break repair/BER components (XRCC1), and the translesion synthesis polymerase POLQ (Figure 4A). Gene set enrichment analysis (GSEA) using GO and KEGG annotations showed negative enrichment of DDR‐related pathways—such as “Homologous Recombination,” “DNA Replication,” “Double‐strand Break Repair,” and “Response to Radiation”—in SIRT7‐deficient cells (Figure 4B). Consistently, GO analysis highlighted DNA repair–related biological processes among the downregulated programs (Figure 4C). Together, these data suggest that SIRT7 depletion compromises double‐strand break (DSB) repair capacity in PanNET cells.

We next validated key findings by RT–qPCR in BON‐1 and QGP‐1 cells exposed to irradiation (IR; 4 Gy), with or without the SIRT7 inhibitor 97491 (10 µM). IR alone increased the expression of MRE11, BRCA1, BRCA2, RAD50, and POLQ. In contrast, SIRT7 inhibition reduced MRE11 and RAD50 in both cell lines, and the combination of IR + 97491 attenuated the IR‐induced upregulation of these genes (Figure 4D,E). Given the role of the MRN complex in initiating DSB signaling, these findings are consistent with the idea that SIRT7 activity supports the transcriptional induction of key DSB repair factors following irradiation [39]. Time‐course analyses further showed that SIRT7 inhibition led to sustained γ‐H2AX accumulation and altered RAD50/MRE11 expression dynamics over 24 h after IR in both cell lines (Figure 4F,G), consistent with impaired DSB repair. To complement pharmacologic inhibition, we performed genetic depletion experiments. Colony formation assays showed that SIRT7 knockdown significantly sensitized BON‐1 and QGP‐1 cells to ionizing radiation (0, 1, 4, and 8 Gy) relative to controls (Figure S4A,B). In line with defective repair, SIRT7‐depleted cells retained more γ‐H2AX foci at 12 and 24 h after IR (Figure S4C,D). Comet assays further confirmed increased DNA damage in the SIRT7 knockdown + IR condition, reflected by a higher tail moment at 12 h post‐IR compared with IR alone (Figure S4E,F). These results mirror the radiosensitizing effects observed with 97491 and support a functional role for SIRT7 in promoting radioresistance in PanNET cells.

Because SIRT7 regulates MEN1 expression and MEN1 has been implicated in DNA damage responses [40], we generated MEN1 knockout (MEN1‐KO) cells using CRISPR–Cas9. In BON‐1 and QGP‐1 cells treated with 97491, MEN1 deletion increased RAD50 and MRE11 protein levels (Figure 4H,I). This suggests that MEN1 status can modulate MRN abundance and thereby influence DSB repair capacity and downstream DDR dynamics. To test whether MEN1 levels quantitatively influence repair capacity and radiosensitivity, we established a doxycycline‐inducible MEN1 re‐expression system in the MEN1‐KO background in BON‐1 and QGP‐1 cells. Increasing doxycycline doses (0, 5, 20, 80, and 320 ng/mL) produced graded MEN1 expression (Figure S5A). RAD50 and MRE11 levels inversely tracked with MEN1 re‐expression, with higher doxycycline doses associated with progressively lower MRN abundance. After 4 Gy IR, γ‐H2AX foci quantified at 12 h post‐IR showed a clear dose‐dependent pattern (Figure S5B,C). Clonogenic assays showed a similar quantitative relationship, with higher MEN1 levels associated with poorer survival after IR (Figure S5D,E). These trends were consistent across both cell lines. Collectively, these data indicate that MEN1 levels quantitatively shape MRN complex abundance, DSB repair signaling, and clonogenic survival following irradiation, supporting MEN1 as a key determinant of radiosensitivity in PanNET cells.

### 2.5 Establishment of a Radioresistant BON‐1 Cell Model Reveals SIRT7 as a Key Mediator of Radioresistance

**FIGURE 5:**
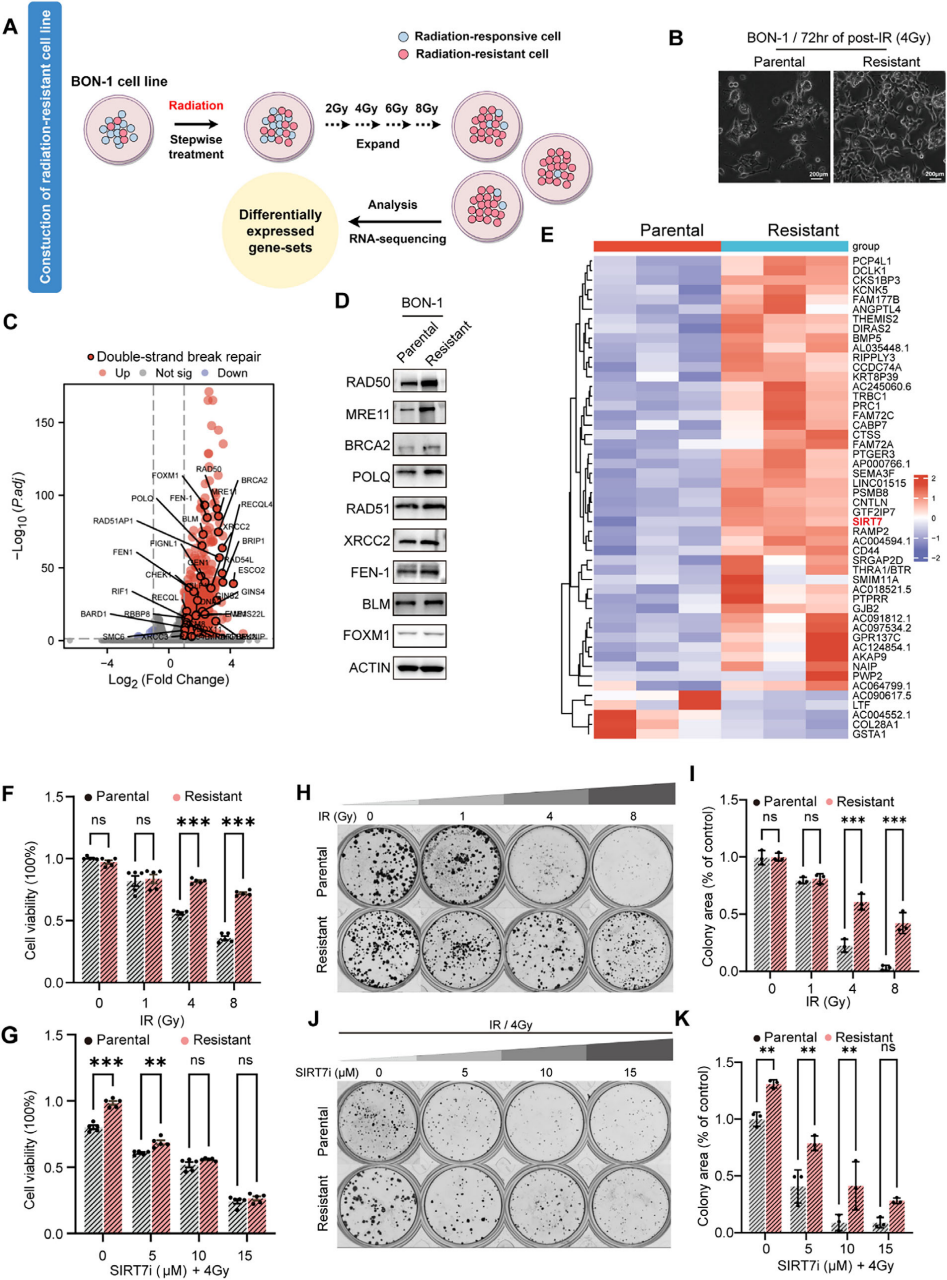
Establishment of a radioresistant BON‐1 model identifies SIRT7 as a mediator of acquired radioresistance. (A) Workflow schematic showing stepwise fractionated irradiation used to generate radioresistant BON‐1 cells (BON‐1/RR) from parental BON‐1 cells, followed by RNA‐seq profiling. (B) Representative phase‐contrast images of parental and resistant BON‐1 cells at 72 h after 4 Gy irradiation. Scale bar, 200 µm. (C) Volcano plot of differentially expressed genes (resistant vs parental BON‐1), with double‐strand break (DSB) repair genes highlighted. *n* = 3 biologically independent samples per group. (D) Immunoblot validation of selected DSB repair factors (RAD50, MRE11, BRCA2, POLQ, RAD51, XRCC2, FEN1, BLM, FOXM1) in parental and resistant BON‐1 cells. *n* = 3 independent experiments. (E) Heatmap of selected differentially expressed genes showing transcriptional upregulation in resistant cells, including SIRT7. *n* = 3 biologically independent samples per group. (F) Cell viability after increasing irradiation doses (0, 1, 4, 8 Gy) comparing parental vs resistant BON‐1 cells. n = 5 biologically independent experiments. Statistics: unpaired two‐sided t‐test. (G) Cell viability of parental versus resistant BON‐1 cells treated with SIRT7 inhibitor (SIRT7i; 0, 5, 10, 15 µM) in combination with 4 Gy irradiation. *n* = 5 biologically independent experiments. Statistics: unpaired two‐sided t‐test. (H) Representative colony formation images of parental and resistant BON‐1 cells after graded irradiation (0, 1, 4, 8 Gy). (I) Quantification of clonogenic growth shown as colony area (% of non‐irradiated control). *n* = 3 biologically independent experiments. Statistics: unpaired two‐sided t‐test. (J) Representative colony formation images of parental and resistant BON‐1 cells after 4 Gy irradiation combined with increasing SIRT7i doses (0, 5, 10, 15 µM). (K) Quantification of colony area (% of 0 µM control). *n* = 3 biologically independent experiments. Statistics: unpaired two‐sided t‐test. Data are presented as mean ± SEM unless otherwise indicated; ns, not significant; *p < 0.05, **p < 0.01, ***p < 0.001.

To investigate mechanisms driving radioresistance in PanNET cells, we established a radioresistant BON‐1 subline (BON‐1/RR) by stepwise fractionated irradiation (Figure 5A). Morphologically, BON‐1/RR cells showed increased tolerance to radiation: 72 h after 4 Gy irradiation, parental BON‐1 cells exhibited extensive cell death and fragmentation, whereas BON‐1/RR cells largely maintained normal morphology with minimal apoptotic features (Figure 5B). To assess phenotypic stability, BON‐1/RR cells were cultured without maintenance irradiation up to passage 20. Clonogenic survival and viability assays at P20 were comparable to those in early‐passage BON‐1/RR cells, and both remained markedly more radioresistant than parental cells (Figure S5F,G). Cell viability assays showed a consistent trend (Figure S5H). These findings indicate that the radioresistant phenotype is functionally stable for at least 20 passages after cessation of irradiation.

RNA‐seq comparing parental and BON‐1/RR cells revealed broad transcriptional differences, with DNA damage response (DDR)‐related genes prominently enriched in the resistant population (Figure 5C). Consistently, GSEA showed significant enrichment of DDR pathways, including Homologous Recombination and DNA Replication (Figure S5I). We validated these findings by RT–qPCR and immunoblotting of key DSB repair factors. RAD50, MRE11, BRCA2, POLQ, RAD51, XRCC2, FEN1, BLM, and FOXM1 were consistently upregulated at both mRNA and protein levels in BON‐1/RR cells relative to parental controls (Figure S5J and Figure 5D), with particularly strong increases in RAD50 and MRE11, consistent with enhanced DSB repair capacity. Among 585 genes significantly upregulated in resistant cells, SIRT7 was markedly overexpressed (Figure 5E), suggesting a potential role in the acquisition of radioresistance.

Functional assays further supported this phenotype: compared with parental cells, BON‐1/RR cells exhibited higher viability (Figure 5F,G) and greater clonogenic survival after irradiation (Figure 5H,I). Importantly, pharmacologic inhibition of SIRT7 resensitized BON‐1/RR cells to radiation, reducing both viability and colony formation following IR (Figure 5J,K). Together, these data establish BON‐1/RR as a stable radioresistant model associated with enhanced DDR pathway activation and identify SIRT7 as a key contributor to the acquired resistant state.

### 2.6 SIRT7 Inhibition Increases the Degree of Radiation‐Induced DNA Damage and Impairs DNA Damage Response

**FIGURE 6:**
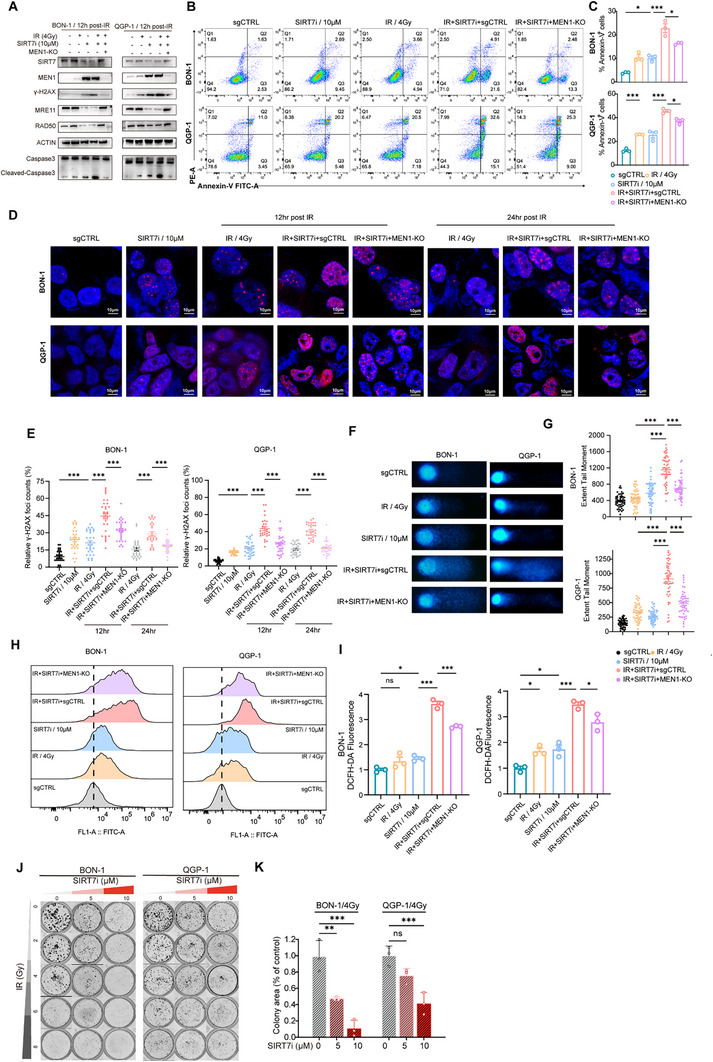
SIRT7 inhibition enhances radiation‐induced DNA damage, oxidative stress, and apoptosis in PanNET cells in a MEN1‐dependent manner. (A) Immunoblot analysis of γ‐H2AX, cleaved caspase‐3, RAD50, MRE11, SIRT7, and MEN1 in BON‐1 and QGP‐1 cells treated with SIRT7 inhibitor (SIRT7i, 10 µM) and/or ionizing radiation (IR, 4 Gy). Cells were harvested 12 h post‐IR. *n* = 3 independent experiments. (B,C) Annexin V/PI flow cytometry apoptosis assays performed 12 h post‐IR in BON‐1 and QGP‐1 cells under the indicated treatments. *n* = 3 biologically independent experiments. Statistics: one‐way ANOVA. (D,E) γ‐H2AX immunofluorescence staining (D) and quantification of γ‐H2AX foci (E) in BON‐1 and QGP‐1 cells at 12 h and 24 h post‐IR under the indicated treatments. Scale bar, 10 µm. *n* = 3 biologically independent experiments; Statistics: one‐way ANOVA at each time point. (F, G) Comet assays in BON‐1 and QGP‐1 cells at 12 h post‐IR under the indicated treatments. Left, representative comet images; right, quantification of tail moment. *n* = 3 biologically independent experiments. Statistics: one‐way ANOVA. (H, I) Intracellular ROS measured by DCFH‐DA staining and flow cytometry in BON‐1 and QGP‐1 cells at 12 h post‐IR under the indicated treatments; representative histograms (H) and quantification (I) are shown. *n* = 3 biologically independent experiments. Statistics: one‐way ANOVA. (J,K) Colony formation assays in BON‐1 and QGP‐1 cells treated with increasing concentrations of SIRT7i (0–10 µM) with or without IR; representative colony images (J) and quantification (K) of colony area are shown. n = 3 biologically independent experiments. Statistics: one‐way ANOVA. Data are presented as mean ± SEM unless otherwise indicated. Significance is shown in the figure; ns, not significant; *p < 0.05, **p < 0.01, ***p < 0.001.

To define the role of SIRT7 in the radiation response, we assessed DNA damage, apoptosis, and cell survival after treatment with the SIRT7 inhibitor 97491 (10 µM), irradiation (IR; 4 Gy), or the combination. Immunoblotting showed that 97491 increased γ‐H2AX and cleaved caspase‐3 in BON‐1 and QGP‐1 cells, with a stronger effect when combined with IR. These changes were markedly attenuated in MEN1‐KO cells (Figure 6A). Notably, although MRE11 and RAD50 levels were reduced under these conditions, γ‐H2AX remained elevated. Given that γ‐H2AX at later time points reflects not only initial DSB sensing but also the persistence of unrepaired lesions and delayed γ‐H2AX resolution, we interpret the increased γ‐H2AX signal as indicative of impaired DSB repair rather than enhanced upstream activation [41].

We also noted that IR increased SIRT7 protein levels, whereas 97491 reduced them. To determine whether these effects reflect transcriptional regulation, we measured SIRT7 mRNA. RT–qPCR showed that IR (4 Gy, 12 h) increased SIRT7 mRNA in both BON‐1 and QGP‐1 cells, whereas 97491 (10 µM, 12 h) decreased SIRT7 mRNA (Figure S6A). We then evaluated protein stability using a cycloheximide (CHX) chase (10 µg/mL, 12 h). Without CHX, IR increased SIRT7 protein, and 97491 decreased it; however, when de novo translation was blocked by CHX, neither treatment altered SIRT7 protein abundance (Figure S6B,C). These results suggest that the observed changes in SIRT7 protein are largely driven by altered transcription rather than major changes in protein stability under these conditions. Consistent with this interpretation, IR is known to activate stress/DDR‐linked transcriptional programs that reshape gene expression following genotoxic stress [42], whereas small‐molecule target inhibition can, in some contexts, reduce target‐gene transcription through feedback or autoregulatory circuitry [43].

Flow cytometry further showed that combined 97491 and IR significantly increased apoptosis compared with either treatment alone, and this increase was substantially reduced in MEN1‐KO cells (Figures 6B,C). Consistently, γ‐H2AX immunofluorescence demonstrated that IR alone induced damage that gradually resolved, whereas addition of 97491 led to sustained γ‐H2AX foci at 12 and 24 h. This persistent damage signal was largely alleviated in MEN1‐KO cells (Figure 6D,E). Comet assays confirmed greater DNA fragmentation with the combination compared with IR alone, again with attenuation in MEN1‐KO cells (Figure 6F,G). We next measured intracellular ROS. DCFH‐DA staining showed that the combination markedly increased ROS, which was significantly reduced in MEN1‐KO cells (Figure 6H,I). In long‐term clonogenic assays, 97491 alone reduced colony formation in a dose‐dependent manner (Figure 6J), and adding IR further decreased clonogenic survival (Figure 6K). The radiosensitizing effect of 97491 was significantly blunted in MEN1‐KO cells. Similar results were observed in patient‐derived PanNET organoids (PDOs): the combination reduced organoid viability and growth more than either treatment alone, whereas siRNA‐mediated MEN1 knockdown partially restored organoid survival (Figure S6D,E). Together, these findings demonstrate that SIRT7 inhibition enhances radiation‐induced DNA damage, oxidative stress, and apoptosis in PanNET cells, and that these radiosensitizing effects are substantially attenuated in the absence of MEN1.

### 2.7 SIRT7 Inhibitor Enhances Radiation‐Mediated Antitumor Effects in Patient‐Derived PanNET Animal Models

**FIGURE 7:**
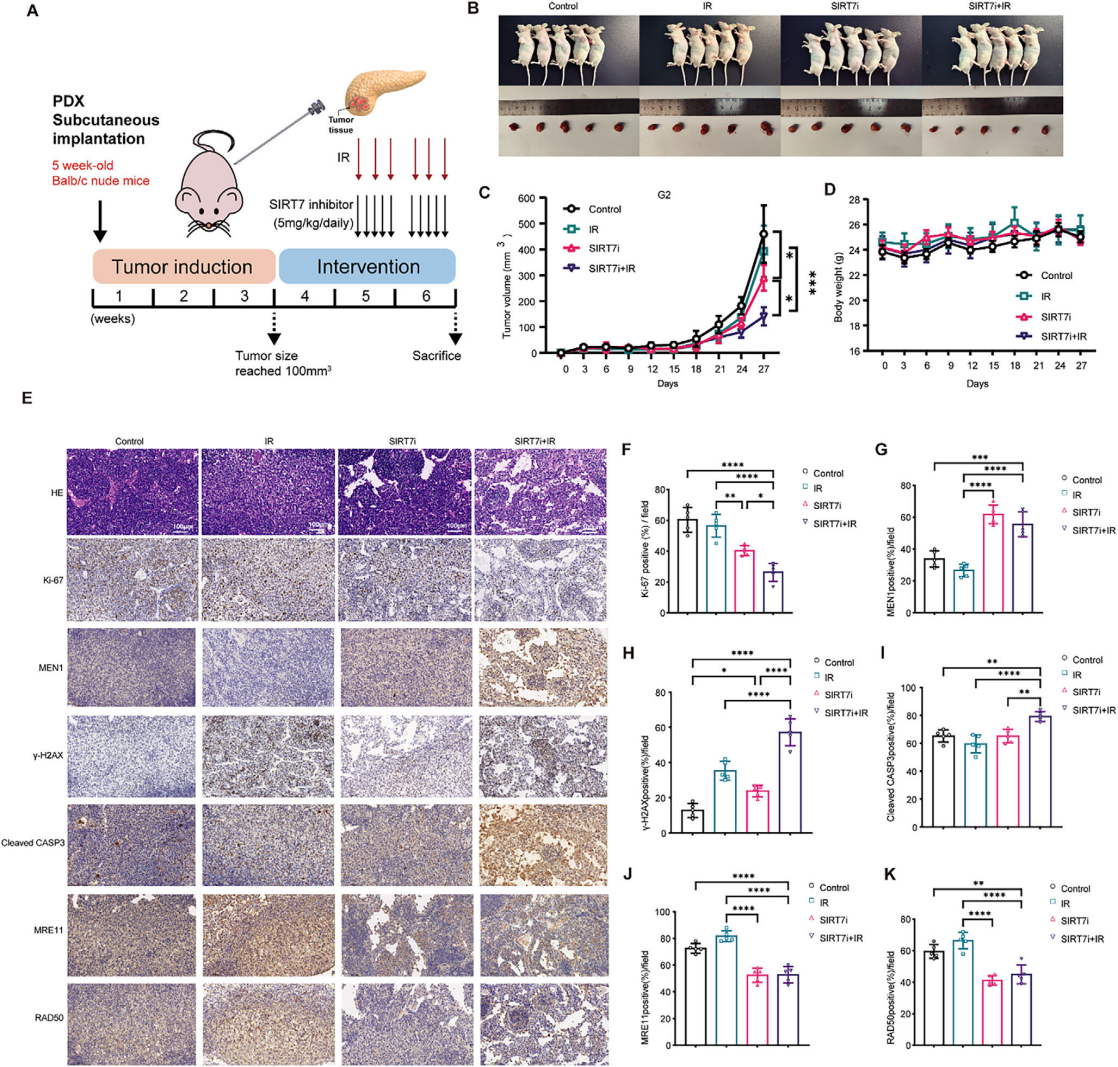
The SIRT7 inhibitor 97491 enhances radiation‐induced antitumor efficacy in PanNET patient‐derived xenograft (PDX) models. (A) Schematic of the PDX study design and treatment schedule. *n* = 5 mice per group. (B) Representative images of excised tumors at endpoint from each group. *n* = 5 mice per group. (C) Tumor growth curves during the treatment period. *n* = 5 mice per group. Statistics: one‐way ANOVA at each timepoint. (D) Body weight monitored throughout the study. *n* = 5 mice per group. Statistics: one‐way ANOVA at each timepoint. (E) Representative H&E and IHC staining of tumor sections for Ki‐67, MEN1, γ‐H2AX, cleaved caspase‐3 (cleaved CASP3), MRE11, and RAD50. Images are shown at 200×; scale bar, 100 µm. (F–K) Quantification of IHC positivity (% positive cells per field) for (F) Ki‐67, (G) MEN1, (H) γ‐H2AX, (I) cleaved CASP3, (J) MRE11, and (K) RAD50. *n* = 5 mice per group; Statistics: one‐way ANOVA. Data are presented as mean ± SEM unless otherwise indicated. Significance is shown in the figure; ns, not significant; *p < 0.05, **p < 0.01, ***p < 0.001.

We established subcutaneous patient‐derived xenograft (PDX) models in five‐week‐old BALB/c nude mice. Three independent PDX lines representing G1, G2, and G3 tumors were generated to assess generalizability. Figure S6F shows primary tumors of patients at different grades (G1, G2, G3). Once tumors reached approximately 100 mm^3^, mice were randomized into four treatment groups: vehicle control, radiation therapy alone (IR; 12 Gy total dose delivered as 2 Gy/fraction, 3 days/week for 2 weeks), SIRT7 inhibitor alone (97491; 5 mg/kg/day, oral gavage, 5 days/week for 2 weeks), or combination therapy (Figure 7A). In all three PDX models, SIRT7 inhibition alone significantly suppressed tumor growth compared with vehicle controls, while IR monotherapy produced a moderate effect. Notably, the combination of SIRT7 inhibition and IR consistently resulted in the greatest tumor growth suppression across all models (Figure 7B,C and Figure S6G,H). Treatment was well tolerated, with no significant body weight loss observed in any group (Figure 7D).

Tumors harvested at endpoint were subjected to IHC analysis. We evaluated proliferation (Ki‐67), apoptosis (cleaved caspase‐3), DNA damage (γ‐H2AX), and DSB repair markers (MRE11 and RAD50) (Figure 7E). The combination group showed the lowest Ki‐67 index and the highest levels of γ‐H2AX and cleaved caspase‐3 across all PDX lines (Figure 7F,H,I). Consistent with impaired repair capacity, MRE11 and RAD50 staining were reduced in the SIRT7 inhibitor group and further decreased in the combination group (Figure 7J,K). Increased MEN1 expression was also observed following SIRT7 inhibition (Figure 7G). No cleaved caspase‐3 staining was detected in adjacent normal tissues across treatment groups. This trend was consistent in the other two G1 and G3 PDX models (Figure S6I,J).

To determine whether DNMT1 directly contributes to the irradiation response in vivo, we established xenografts using DNMT1‐knockdown BON‐1 cells and matched vector controls (Figure S7A). Mice bearing DNMT1‐KD tumors were treated with or without IR. DNMT1 knockdown enhanced the antitumor effect of irradiation, with the DNMT1‐KD + IR group showing the smallest tumor volumes compared with IR alone or DNMT1‐KD alone (Figure S7B). Consistently, IHC analysis demonstrated increased γ‐H2AX and cleaved caspase‐3 staining together with reduced Ki‐67 in the DNMT1‐KD + IR group (Figure S7C–I). These findings suggest that DNMT1 plays a functional role in modulating tumor response to irradiation in vivo.

Overall, these results show that SIRT7 inhibition enhances radiation‐mediated antitumor effects across multiple independent PanNET PDX models and that DNMT1 plays a functional role in the irradiation response in vivo.

## 3 Discussion

MEN1 is the most frequently mutated gene in PanNETs and plays a pivotal roles in multiple cellular pathways. Our recent multi‐omics study involving proteomic profiling of 108 nonfunctional PanNETs provided insight into MEN1 alterations and identified mediators of MEN1‐driven tumorigenesis [12]. However, many regulatory mechanisms underlying MEN1 function remain unclear. Here, we demonstrate that SIRT7 physically interacts with DNMT1 and promotes catalytic activity–dependent enrichment of DNMT1 at the MEN1 promoter, thereby driving promoter methylation and transcriptional silencing. This finding contributes to the evolving understanding of epigenetic regulation and expands the known functions of the versatile sirtuin family.

SIRT7 is a multifunctional sirtuin implicated in tumor cell stress resistance and immune evasion. For example, in melanoma SIRT7 enhances stress resilience and upregulates PD‐L1 by deacetylating SMAD4 to relieve IRE1α repression [28]. It also employs diverse chromatin regulatory strategies to coordinate tumor stress adaptation by controlling metabolism‐ and immune‐related gene expression [44]. Beyond its well‐known deacetylase activity, SIRT7 catalyzes mono‐ADP‐ribosylation [45] and desuccinylation [46]. For instance, SIRT7‐mediated desuccinylation of PRMT5 at K387 increases PRMT5 methyltransferase activity and maintains epigenetic homeostasis [47]. SIRT7 can also influence targets through non‐enzymatic means, as in lung cancer it destabilizes the tumor suppressor ARF via direct protein–protein interaction [48]. In our study, we found that SIRT7 functions not merely as a passive scaffold but as an enzymatic regulator that establishes a chromatin environment permissive for DNMT1‐mediated repression.

A pivotal finding of our study is that disrupting the SIRT7/DNMT1‐MEN1 regulatory axis impairs DDR capacity in PanNETs. This is noteworthy because robust tumor DDR mechanisms contribute to intrinsic or acquired radioresistance, as exemplified by the limited efficacy of ^1^ ^7^ ^7^Lu‐DOTATATE peptide receptor radionuclide therapy (PRRT) in some advanced gastroenteropancreatic NETs (NETTER‐2 trial) [49]. Notably, efforts to augment radiotherapy by targeting DDR are showing promise. Reuvers et al. demonstrated that the DNA‐PKcs inhibitor AZD7648 significantly enhances PRRT efficacy in SSTR2‐expressing NET models by blocking double‐strand break repair [50]. Likewise, Riaz et al. reported that ATR inhibitor RP‐3500 produced synergy with radiotherapy in ATM‐null tumors [51]. Combining DDR‐targeted therapies with other treatments has also achieved clinical success. The PARP inhibitor talazoparib plus enzalutamide (TALAPRO‐2 trial) was recently FDA‐approved for HRR gene‐mutated metastatic castration‐resistant prostate cancer [52]. Furthermore, the DUO‐O trial (NCT03737643) demonstrated that adding durvalumab plus olaparib maintenance after chemotherapy improved progression‐free survival in HR‐deficient ovarian cancer patients, validating the concept that PARP inhibition can increase genomic instability and enhance neoantigen presentation. These examples suggest that stratifying patients by the status of DDR pathways could guide therapy selection.

Radiation‐induced DNA damage largely determines radiosensitivity, and PanNETs exhibit heterogeneous DNA repair capacities [40]. Since most radiation‐induced DNA breaks are repaired within 24 h [53], understanding these repair processes is crucial for improving radiotherapy outcomes. Our data indicate that SIRT7 inhibition reduces MRN complex abundance through MEN1 re‐expression, leading to sustained γ‐H2AX accumulation, increased apoptosis, and impaired clonogenic survival. This finding aligns with prior reports that SIRT7 has broad involvement in the DDR. SIRT7 is recruited to DNA break sites via PARP1‐dependent mechanisms [54], deacetylates ATM at K3016 to modulate ATM activity [27], and deacetylates histone H3K18 to promote 53BP1 recruitment during nonhomologous end joining [26]. Based on these observations, we propose a refined model in which SIRT7 influences DDR in PanNETs through two coordinated mechanisms: (1) transcriptional repression of MEN1 via DNMT1‐dependent promoter methylation, thereby modulating MRN complex expression, and (2) modulation of DNA repair protein activity via posttranslational mechanisms described in other tumor types. This dual function may contribute to intertumoral heterogeneity in radiosensitivity.

**FIGURE 8:**
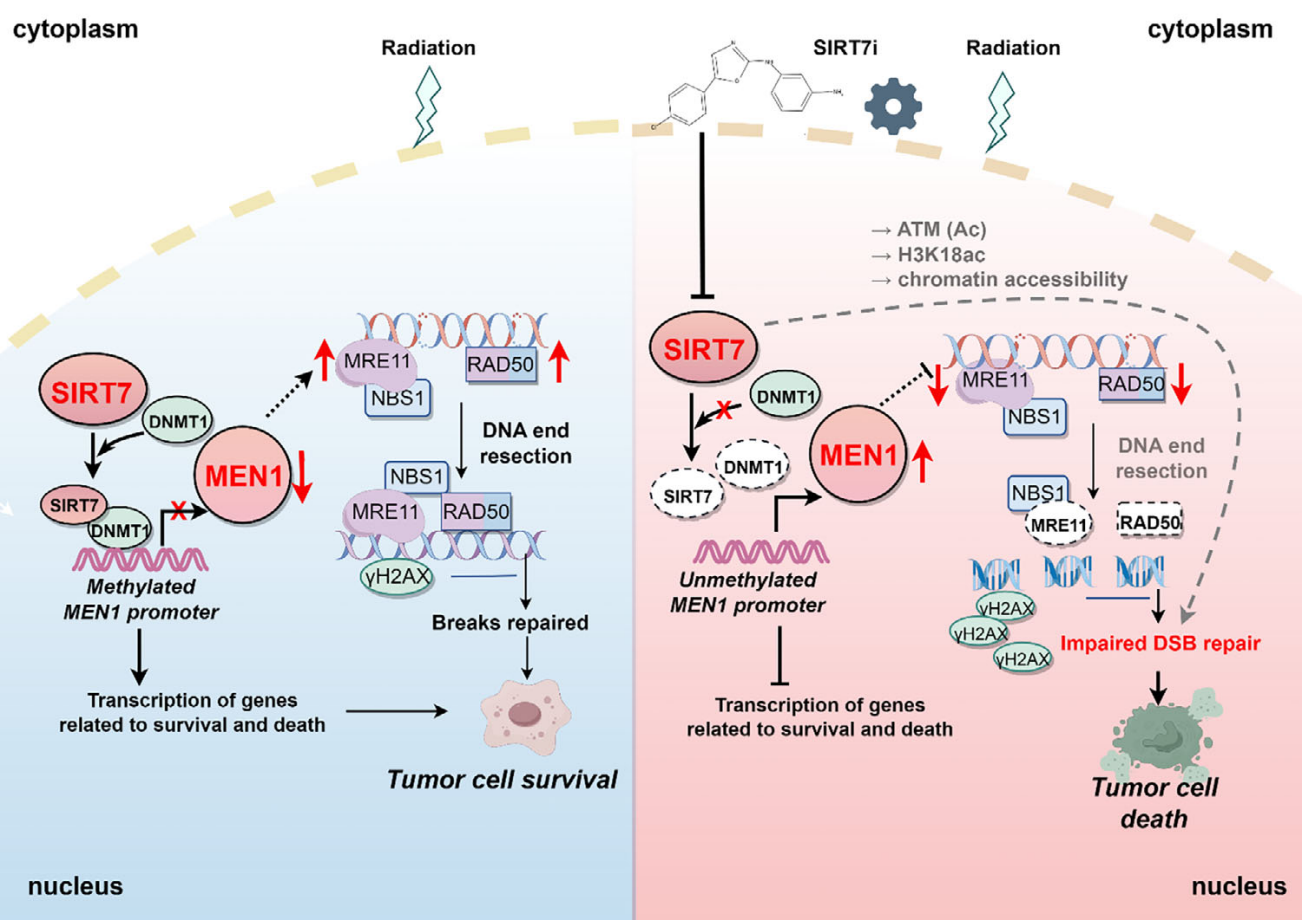
The proposed working model of this study. SIRT7 recruits DNMT1 to the MEN1 promoter, leading to promoter methylation and repression of MEN1 transcription. Reduced MEN1 expression promotes MRN complex activity and efficient DNA repair following irradiation, contributing to tumor cell survival. Pharmacologic inhibition of SIRT7 restores MEN1 expression, reduces MRN complex abundance, impairs DSB repair, and enhances radiosensitivity. In addition to MEN1‐dependent regulation, SIRT7 may also modulate DNA damage responses through parallel MEN1‐independent mechanisms (e.g., ATM acetylation and chromatin accessibility changes), which converge on impaired DSB repair and tumor cell death. DSB: DNA double‐strand break.

Consistent with its broad influence, MEN1 orchestrates several critical cellular pathways in neuroendocrine neoplasms and serves as a central hub in the DNA repair network [11, 55, 56, 57]. In our study, graded re‐expression experiments demonstrated that MEN1 levels quantitatively influence MRN complex abundance, DNA repair efficiency, and radiosensitivity, strengthening the causal link between MEN1 suppression and DDR regulation. Furthermore, in vivo DNMT1 knockdown enhanced radiation responses, supporting a functional role for DNMT1 within this regulatory axis beyond correlative epigenetic observations. Our findings show that SIRT7/DNMT1‐mediated MEN1 downregulation impairs the MRN complex (MRE11–RAD50–NBS1; see working model in Figure 8), whose deficiency is associated with hypersensitivity to ionizing radiation [58]. This builds on our previous work showing that MEN1 overexpression promotes β‐TrCP binding to β‐catenin, elevating MGMT levels and increasing tumor sensitivity to temozolomide (TMZ) [34]. Moreover, β‐TrCP has been reported to interact with the MRN complex, recruiting it to DNA breaks via nondegradative ubiquitin signals [59]. These findings suggest that MEN1 may regulate MRN activity through multiple molecular intermediates.

Nevertheless, MEN1 can exhibit paradoxical tumor‐suppressive or oncogenic roles depending on the tumor microenvironment [60]. For example, in an in vivo CRISPR screen, MEN1 knockout redistributed MLL1 chromatin occupancy, increasing H3K4me3 at repetitive elements and activating viral mimicry pathways (MAVS and cGAS–STING). This epigenetic reprogramming triggered an immunogenic phenotype that enhanced anti–PD‐1 therapy efficacy, illustrating how epigenetic changes can convert “cold” tumors into “hot” ones. Given this duality of MEN1, future work should examine tissue‐specific roles of the SIRT7/DNMT1‐MEN1 axis across distinct epigenetic and immune landscapes.

Several points should be considered when interpreting our findings. While we center on SIRT7/DNMT1‐mediated MEN1 repression, we do not exclude MEN1‐independent functions of SIRT7 in DDR regulation, as reported in other malignancies [20]. In addition, although our data do not support a major role for SIRT5 in MEN1 regulation in PanNETs, more subtle or context‐dependent effects cannot be completely excluded. Moreover, while our data strongly support a locus‐specific mechanism at the MEN1 promoter, they do not define the broader extent of SIRT7‐mediated chromatin regulation in PanNETs. Although 97491 produced effects consistent with SIRT7 inhibition in our assays, the potential limitations and possible off‐target effects of this pharmacological inhibitor should be considered when interpreting these results.

Despite these considerations, integration of spatial transcriptomics, validated biomarkers, and precision dosimetry may enable patient selection based on SIRT7–DNMT1–MEN1 axis activity. Furthermore, combining SIRT7 inhibition with DDR‐targeted agents or PRRT may provide a rational strategy to enhance therapeutic efficacy. In conclusion, our study defines a catalytic activity–dependent SIRT7–DNMT1–MEN1 axis that regulates radiosensitivity in pancreatic neuroendocrine tumors. By linking epigenetic silencing of MEN1 to impaired MRN‐mediated DNA repair and sustained radiation‐induced damage, we identify a mechanistically grounded vulnerability that may inform precision radiotherapy strategies in PanNETs.

## 4 Experimental Section

### 4.1 Cell Lines and Reagents

Human pancreatic neuroendocrine tumor (PanNET) cell lines BON‐1 (RRID: CVCL_3985) and QGP‐1 (RRID: CVCL_3143) were used. BON‐1 cells were a kind gift from Prof. Martyn Caplin (Royal Free Hospital, UK), and QGP‐1 cells were obtained from Shanghai Zhong Qiao Xin Zhou Biotechnology. Cells were grown in DMEM/F12 (BON‐1) or RPMI‐1640 (QGP‐1) medium supplemented with 10% fetal bovine serum (FBS) and 1% penicillin–streptomycin, at 37°C in a humidified atmosphere with 5% CO_2_. All cell lines were authenticated by short tandem repeat (STR) profiling and regularly tested to be mycoplasma‐free prior to use.

Small‐molecule inhibitors were purchased from Selleck Chemicals and used according to the manufacturer's instructions. Unless otherwise specified, each compound was applied at two doses, defined as low (L) and high (H), as follows:

HDAC5 inhibitor (LMK‐235, Cat# S7569) – L: 1.5 nm; H: 15 nm
SIRT2 inhibitor (SirReal2, Cat# S7845) – L: 1 µ; H: 10 µm
SIRT3 inhibitor (3‐TYP, Cat# S8628) – L: 2 µm; H: 20 µm
SIRT5 inhibitor (NRD167, Cat# S9903) – L: 1 µm; H: 10 µm
SIRT7 inhibitor (97491, Cat# E1150) – L: 1 µm; H: 10 µm

Additionally, DNA methyltransferase 1 (DNMT1) inhibitors 6‐thioguanine (6‐TG, Cat# S1774) and 5‐azacytidine (5‐AZA, Cat# S1782) were obtained from Selleck and used at the concentrations indicated in the text.

### 4.2 CRISPR Knockout Library Screening and Data Analysis

BON‐1 cells were first transduced with a lenti‐Cas9‐Blast vector (Addgene) at a multiplicity of infection (MOI) < 0.7 and selected with blasticidin to establish a stable Cas9‐expressing line. These Cas9‐positive cells were then infected with a pooled lentiviral single‐guide RNA (sgRNA) library (Yomebio, Scishare R162256) at MOI < 0.3. The library targeted 2,508 epigenetic regulators using 20,051 unique sgRNAs (approximately 8 guides per gene). After puromycin selection, library coverage of >300× was maintained throughout the screen to ensure representation of all sgRNAs.

At 8 and 10 days post‐infection, approximately 5 × 10^7^ cells per replicate were harvested and processed for MEN1 protein level detection using an intracellular immunofluorescence staining protocol as described in [30]. Cells were fixed with 4% paraformaldehyde for 15 min and permeabilized with 0.1% Triton X‐100 for 20 min at room temperature. After two washes with ice‐cold PBS, cells were blocked with 5% bovine serum albumin (BSA) in PBS for 1 h. Next, cells were incubated with primary antibody (rabbit anti‐MEN1, Boster Biological Technology, Cat# A00331‐1; 1 µg per 1 × 10^6^ cells) for 30 min at room temperature, followed by a DyLight 488–conjugated goat anti‐rabbit IgG secondary antibody (Boster, Cat# BA1127; 5–10 µg per 1 × 10^6^ cells) for 30 min at room temperature in the dark. Labeled cells were filtered through a 40‐µm cell strainer to remove clumps and then subjected to fluorescence‐activated cell sorting (FACS). The FACS was used to isolate two populations based on MEN1 fluorescence intensity: a MEN1‐low fraction (bottom 50% of cells) and a MEN1‐high fraction (top 10% of cells).

Genomic DNA was extracted from the sorted cell populations, and sgRNA sequences were PCR‐amplified using a one‐step CRISPR NGS Library Construction Kit (Yomebio, Cat# PK201). Amplicons were deep sequenced on an Illumina NovaSeq platform to determine sgRNA representation in each fraction. For data analysis, sequencing reads were aligned to the reference sgRNA library, and read counts were normalized across samples with a small pseudocount added to zero values. Log_2_ fold‐changes in sgRNA abundance between the MEN1‐high and MEN1‐low fractions were calculated. Gene‐level enrichment scores were then generated using the MAGeCK MLE algorithm [31]. This analysis identified epigenetic regulators whose CRISPR/Cas9 knockout significantly altered MEN1 protein levels in PanNET cells.

### 4.3 Study Population and Tissue Microarrays

We retrospectively enrolled 121 patients with pathologically confirmed PanNET who underwent surgical resection at Fudan University Shanghai Cancer Center between June 2012 and January 2022. The study was conducted under Institutional Ethics Committee approval (Approval No. 2105235), in accordance with the Declaration of Helsinki. Informed consent was obtained from all patients. For a subset of cases (*n* = 70), paired tumor and adjacent normal tissue specimens were used to construct tissue microarrays (TMAs). Clinical and pathological characteristics of this patient cohort, including outcomes, have been reported previously [34]. Disease‐free survival (DFS) was defined as the time from surgery until documented disease progression or recurrence.

Kaplan–Meier survival curves were generated to evaluate the association between SIRT7 expression and DFS, and differences between groups were assessed using the log‐rank test. Cox proportional hazards regression models were applied to estimate hazard ratios (HRs) and corresponding 95% confidence intervals (CIs). The proportional‐hazards assumption was formally tested using a standard diagnostic method, and no violation of the assumption was detected (*χ* ^2^ = 0.267, df = 1, *p* = 0.605).

### 4.4 Western Blot Analysis

Protein expression in cell lysates was examined by Western blotting. Equal amounts of total protein were resolved by SDS‐PAGE and transferred to PVDF membranes. Membranes were probed with primary antibodies specific to the following proteins: MEN1 (BBI, Cat# D263072), SIRT7 (ABclonal, Cat# A21731), DNMT1 (ABclonal, Cat# A16729), MRE11 (ABclonal, Cat# A4222), RAD50 (ABclonal, Cat# A3078), γ ‐ H2AX (ABclonal, Cat# AP0687), Caspase‐3 and Cleaved Caspase‐3 (Proteintech, Cat# 66470‐2‐Ig), and Flag epitope tag (ABclonal, Cat# AE005). ACTIN (ABclonal, Cat# AC038) was used as a loading control. After incubation with appropriate horseradish peroxidase (HRP)‐conjugated secondary antibodies, signals were detected using an enhanced chemiluminescence system and quantified by densitometry.

### 4.5 TMA Immunohistochemistry (IHC)

#### 4.5.1 Staining

Paraffin‐embedded TMA sections were subjected to immunohistochemical staining for various biomarkers. Slides were deparaffinized, rehydrated, and underwent antigen retrieval (in sodium citrate buffer, pH 6.0, at 95°C for 15 min). After blocking endogenous peroxidase and non‐specific binding, sections were incubated with primary antibodies against MEN1 (BBI, Cat# D263072), Ki‐67 (Abcam, Cat# ab16667), γ ‐ H2AX (ABclonal, Cat# AP0687), RAD50 (ABclonal, Cat# A0182), MRE11 (ABclonal, Cat# A2559), or Cleaved Caspase‐3 (Proteintech, Cat# 25128‐1‐AP). All antibodies were used at a 1:200 dilution in antibody diluent and incubated for 1 h at room temperature. After washing, slides were incubated with HRP‐linked secondary antibodies for 30 min, developed with DAB substrate, and counterstained with hematoxylin. Staining results were evaluated by two independent pathologists. Protein expression levels in tumor cells were semi‐quantitatively scored based on staining intensity and the percentage of positive cells, and then dichotomized into “high” versus “low” expression groups using the cohort median as the cutoff.

#### 4.5.2 Scoring and Quantification

Immunohistochemical (IHC) staining was evaluated using a semi‐quantitative scoring system (modified H‐score/IHC score). Two independent, board‐certified pathologists, blinded to the clinical and pathological information, assessed marker staining in all tissue samples. Each specimen was scored based on: (1) the percentage of positive cells (0%, 0%; 1%, 1%–25%; 2%, 26%–50%; 3%, 51%–75%; 4%, 76%–100%); and (2) staining intensity (0, none; 1, weak; 2, moderate; 3, strong). The final IHC score was calculated as the product of the percentage score and intensity score (score = percentage score × intensity score), yielding a range of 0–12, with higher scores indicating stronger protein expression. In cases of discrepancy, slides were jointly reviewed to reach consensus. Scores were used for subsequent statistical analyses.

### 4.6 Quantitative Analysis of Protein Colocalization

Colocalization between two proteins in immunofluorescence images was quantified using the JaCoP plugin in ImageJ/Fiji [61]. Two complementary parameters were calculated: Pearson's correlation coefficient (PCC) and Manders’ overlap coefficient (M1, M2). PCC reflects the correlation between fluorescence intensities in the two channels across pixels and ranges from –1 to +1. A value close to +1 indicates strong positive correlation, 0 indicates no correlation, and −1 indicates inverse correlation. M1 and M2 evaluate the extent to which the fluorescence signal of one protein spatially overlaps with that of the other, independent of signal intensity correlation. Values approach 1 when the two signals substantially overlap and approach 0 when overlap is minimal. Together, these metrics provide a quantitative assessment of the spatial relationship between the two proteins within the analyzed images.

### 4.7 RNA Sequencing and Analysis

Total RNA was extracted using TRIzol reagent (Invitrogen) according to the manufacturer's protocol. RNA quantity and integrity were assessed using an Agilent 2100 Bioanalyzer, and only high‐quality samples were used for downstream library preparation. Sequencing libraries were constructed using a poly(A) enrichment protocol and sequenced on an Illumina NovaSeq 6000 platform to generate 150‐bp paired‐end reads. Clean reads were aligned to the human reference genome (GRCh38/hg38), and gene‐level read counts were obtained for downstream analysis. Gene detection sensitivity was evaluated using an expression threshold of FPKM ≥ 1. Differential gene expression analysis was performed using DESeq2 in R. Genes with an adjusted *p* value (Benjamini–Hochberg false discovery rate, FDR) ≤ 0.05 were considered significantly differentially expressed. Functional enrichment analyses, including Gene Ontology (GO) and KEGG pathway analyses, were conducted on differentially expressed gene sets. Principal component analysis (PCA) was performed to assess sample clustering and reproducibility.

### 4.8 Chromatin Immunoprecipitation (ChIP) and PCR

ChIP assays were carried out using a ChIP kit (Cell Signaling Technology, Cat# 9003) according to the manufacturer's instructions. In brief, PanNET cells were cross‐linked with 1% formaldehyde for 10 min at room temperature to fix protein–DNA complexes, then quenched with 125 mM glycine. Cells were lysed in swelling buffer to isolate nuclei, and chromatin was sheared by sonication to an average size of 200–500 bp. The chromatin lysate was incubated overnight at 4°C with 20 µg of anti‐SIRT7 antibody (Santa Cruz Biotechnology, Cat# sc‐365344) or a 1:50 dilution of anti‐DNMT1 antibody (Cell Signaling, Cat# 5032). Normal rabbit IgG was used as a negative control. Immune complexes were captured by adding 25 µL of Protein G magnetic beads (Cell Signaling, Cat# 9006) for 2 h at 4°C. Beads were then sequentially washed (three times each) with low‐salt wash buffer, high‐salt wash buffer, and LiCl wash buffer to remove non‐specific binding. Chromatin was eluted from the beads and reverse cross‐linked to free the DNA. The primers targeting the MEN1 promoter (P1 region; approximately −81 bp relative to the TSS) were: MEN1 promoter forward, 5′‐GCCAATCCCTGAGTATCTC‐3′ and reverse, 5′‐CGCTGAGCCTGGAAATAG‐3′. Enrichment of DNMT1 at the MEN1 promoter was calculated relative to input DNA and compared to IgG control immunoprecipitations.

### 4.9 Dual‐Luciferase Reporter Assay

For promoter activity assays, PanNET cells were co‐transfected with a series of firefly luciferase reporter plasmids containing different truncated fragments of the MEN1 promoter cloned upstream of the luciferase gene. The promoter constructs included fragments spanning −1000/+250, −750/+250, −500/+250, −250/+250, −80/+120, and −1/+250 relative to the transcription start site (TSS), as described in Figure 1H. A Renilla luciferase control plasmid pRL‐TK was co‐transfected for internal normalization. Transfections were performed using Lipofectamine 3000 (Invitrogen) following the manufacturer's protocol. At 48 h post‐transfection, cells were lysed in Passive Lysis Buffer (Promega), and 10–20 µL of each lysate was transferred to a 96‐well plate for measurement. Firefly and Renilla luciferase activities were sequentially measured using the Dual‐Luciferase Reporter Assay System kit (Promega, Cat# E1910) in the CentroXS LB960 lumiometer (Berthold Technologies). Firefly luminescence was normalized to Renilla luminescence for each sample. Relative luciferase activity was expressed as a fold‐change compared to the control conditions.

### 4.10 Plasmid Transfection and RNA Interference

Stable knockdown of SIRT7 and DNMT1 in BON‐1 and QGP‐1 cells was achieved using the pLKO.1‐puro lentiviral vector (Addgene plasmid #10878), which produces stable knockdown (KD) through lentiviral‐mediated transduction. Two different shRNA constructs were used for each gene:

SIRT7‐Kd#1: Gaactgggaagcggcgaccg
SIRT7‐Kd#2: Gcagctgggtcccacacttg
DNMT1‐Kd#1: Aaatactccgactacatcaa
DNMT1‐Kd#2: Gccaattcggtagggctcag

Lentiviral particles were produced in HEK293T cells and used to transduce PanNET cells. Infected cells were selected with puromycin (2 µg/mL) to establish stable cell pools with SIRT7 or DNMT1 knockdown. For ectopic overexpression of SIRT7, the full‐length human SIRT7 coding sequence was cloned into the pCDH‐CMV‐MCS‐EF1α‐Puro lentiviral vector (System Biosciences) to create the pCDH‐SIRT7 expression construct. After lentiviral transduction with pCDH‐SIRT7 or the empty vector, cells were selected with puromycin to generate stable SIRT7‐overexpressing or control cell lines.

For transient gene silencing, small interfering RNAs (siRNAs) were transfected into cells using Lipofectamine 3000 (Invitrogen) according to the manufacturer's instructions. All siRNAs were purchased from RiboBio (Guangzhou, China). The siRNA target sequences (sense strand, 5′–3′) were:

MEN1 siRNA: GGGAAGACGAGGAGAUCUACA
HDAC1 siRNA: CGUUCUUAACUUUGAACCAUA
HDAC2 siRNA: AAGCCUCAUAGAAUCCGCAUG
HDAC3 siRNA: CCCGCAUCGAGAAUCAGAAUU
HDAC4 siRNA: GUCCAGGCUAAAGCAGAAA
HDAC5 siRNA: GGAGAGCUCAAGAAUGGAUU
HDAC6 siRNA: GCUGCACCGUGAGAGUUCCAA
SIRT1 siRNA: CAGUGUCAUAUCAUCCAACUU
SIRT2 siRNA: GCCAACCAUCUGUCACUACUU
SIRT3 siRNA: GAAACUACAAGCCCAACGUTT
SIRT5 siRNA: GCUGGAGGUUAUUGGAGAA
SIRT7 siRNA: CCAGCCUGAAGGUUCUAAA
Control siRNA (non‐targeting): UUCUCCGAACGUGUCACGUTT

Knockdown or overexpression efficiencies were verified by Western blot and/or qRT‐PCR in each case before proceeding with further assays.

### 4.11 Knockdown Efficiency Validation

Knockdown efficiency of SIRT7 and DNMT1 was quantified at both mRNA and protein levels. For mRNA quantification, total RNA was extracted using the RNeasy kit (Qiagen) and cDNA synthesis was performed using the iScript cDNA Synthesis Kit (Bio‐Rad). RT‐qPCR was carried out using specific primers for SIRT7, DNMT1, and ACTIN (used as a reference gene). The relative mRNA levels were calculated using the ΔΔCt method. For protein analysis, cells were lysed in RIPA buffer, and band intensities were quantified by densitometry using ImageJ software. Protein levels were normalized to the corresponding loading control (ACTIN or HSP90), and knockdown efficiency was expressed as percentage reduction relative to control cells.

At the mRNA level, SIRT7‐KD#1 and #2 in BON‐1 cells achieved 60% and 55% knockdown, respectively, while in QGP‐1 cells, SIRT7‐KD#1 and #2 achieved 45% and 35% knockdown (Figure S1I). At the protein level, SIRT7‐KD#1 and #2 in BON‐1 cells showed 64% and 50% reduction, respectively, and in QGP‐1 cells showed 63% and >90% reduction (Figure 1E). For DNMT1, at the mRNA level, DNMT1‐KD#1 and #2 in BON‐1 cells achieved 50% and 65% knockdown, respectively, while in QGP‐1 cells achieved 45% and 40% knockdown (Figure S3A). At the protein level, DNMT1‐KD#1 and #2 in BON‐1 cells showed approximately 80% reduction, and in QGP‐1 cells showed approximately 70% reduction (Figure 3G). All protein knockdown efficiencies were quantified from ≥3 independent biological experiments.

### 4.12 Co‐Immunoprecipitation and LC–MS/MS Analysis

To identify SIRT7‐interacting proteins, co‐immunoprecipitation followed by mass spectrometry was performed. QGP‐1 cells stably expressing Flag‐tagged SIRT7 were lysed in ice‐cold NP‐40 lysis buffer (50 mM Tris‐HCl pH 7.5, 150 mM NaCl, 1% NP‐40, plus protease and phosphatase inhibitors). For stringent interaction validation, clarified lysates were treated with RNase A (100 µg/mL) and DNase I (50 U/mL) for 30 min at 37°C prior to immunoprecipitation. Where indicated, co‐immunoprecipitation was performed under high‐salt conditions (300 mM NaCl) to minimize potential chromatin‐ or nucleic acid‐mediated associations. Lysates were then incubated with 5 µg of anti‐Flag antibody (Sigma, M2 clone) or normal IgG (control) for 2 h at 4°C with gentle rotation. Subsequently, 10 µL of Protein A/G agarose bead slurry was added to each sample and incubation continued for an additional 1 h at 4°C. The beads were washed three times with lysis buffer, and bound proteins were eluted by boiling in SDS sample buffer. Eluates were separated by SDS‐PAGE and visualized by silver staining. Each lane was excised and subjected to in‐gel tryptic digestion. The resulting peptides were analyzed using a Thermo Fisher Scientific Orbitrap Fusion Lumos mass spectrometer. Tandem mass spectra were searched against the human RefSeq protein database for protein identification.

### 4.13 Pyrosequencing Analysis of MEN1 Promoter Methylation

Genomic DNA was extracted from fresh‐frozen tissues or cultured cells using the QIAamp DNA Mini Kit (QIAGEN) according to the manufacturer's instructions. Bisulfite conversion of 500 ng genomic DNA was performed using the EpiTect Bisulfite Kit (QIAGEN), following the manufacturer's protocol. A 620‐bp transcription start site (TSS)‐centered region of the human MEN1 core promoter was analyzed. Three pyrosequencing amplicons were designed to cover the proximal promoter region. For CpG‐resolved quantitative analysis, nine CpG sites located within the −67 to −2 bp region relative to the TSS were selected for detailed methylation measurement. Genomic coordinates and primer sequences are provided in Table S8.

As shown in Table S11, PCR amplification was performed using bisulfite‐specific primers, and the resulting 169‐bp amplicons were subjected to pyrosequencing on a QIAGEN PyroMark Q48 instrument using PyroMark Q48 software (v2.0). Methylation percentages at each CpG site were calculated automatically by the instrument software and manually reviewed for quality control. Only reads with ≥100 valid signal counts (coverage) per CpG position were included in the analysis. Bisulfite conversion efficiency was validated by confirming complete C‐to‐T conversion of non‐CpG cytosines (>99% efficiency). Fully methylated and unmethylated commercial control DNA samples were included in each experimental batch to confirm assay specificity and sensitivity. Samples showing incomplete bisulfite conversion were excluded from analysis. Each sample was analyzed in three independent technical replicates, including independent bisulfite conversion, PCR amplification, and pyrosequencing runs. The mean methylation value across the nine CpG sites was used for statistical analysis. Reproducibility across replicates showed a coefficient of variation (CV) <5%.

### 4.14 Cell Proliferation and Clonogenic Survival Assays

For cell proliferation assays, cells were seeded at 2.5 × 10^3^ cells per well in 96‐well plates (five replicate wells per condition). Cell growth was measured at indicated time points using the Cell Counting Kit‐8 (CCK‐8, Beyotime, Cat# C0042) according to the manufacturer's protocol; absorbance at 450 nm (which correlates with viable cell number) was recorded with a microplate reader. In addition, DNA synthesis was evaluated using a 5‐ethynyl‐2′‐deoxyuridine (EdU) incorporation assay (Beyotime EdU Kit, Cat# C0071S). For the EdU assay, cells were incubated with EdU for 2 h, fixed, and stained, and EdU‐positive cells were detected by fluorescence microscopy. The percentage of EdU‐positive cells was calculated from five random fields per sample.

For clonogenic survival assays, cells were plated in 6‐well plates at a low density (1000–2000 cells per well) and allowed to grow for ∼12 days to form colonies. Colonies were then fixed with 4% paraformaldehyde for 15 min, stained with 0.1% crystal violet for 30 min, and gently washed with water. Visible colonies were counted manually or with imaging software. Colony formation efficiency was expressed as the fraction of seeded cells that formed colonies, relative to control conditions.

### 4.15 Establishment and Validation of Radioresistant BON‐1 Cells

Radioresistant BON‐1 cells (BON‐1/RR) were established using a stepwise dose‐escalation fractionated irradiation protocol with a cumulative dose of 60 Gy [62, 63, 64, 65]. Exponentially growing BON‐1 cells were irradiated using an X‐ray irradiator (SHARP system) at a dose rate of approximately 1 Gy/min. The fractionation schedule consisted of 12 fractions: 2 Gy × 3 (6 Gy), 4 Gy × 3 (12 Gy), 6 Gy × 3 (18 Gy), and 8 Gy × 3 (24 Gy), reaching a total cumulative dose of 60 Gy. Between fractions, cells were allowed to recover for 2–3 days until reaching 70%–80% confluence before the next irradiation. Fresh complete medium was replaced prior to each fraction to ensure irradiation of actively proliferating cells. Parental BON‐1 cells were cultured in parallel under identical conditions without irradiation to serve as age‐matched controls [66].

After completion of 60 Gy, irradiation was discontinued and BON‐1/RR cells were maintained under standard culture conditions. Early‐passage cells (P3–P5 post‐irradiation) were cryopreserved to establish a master cell bank. To assess phenotype stability, BON‐1/RR cells were cultured without maintenance irradiation up to passage 20 [67]. Radioresistance was evaluated at early (P5) and late (P20) passages using cell viability and clonogenic assays [68] (Figure S5F–H). BON‐1/RR cells consistently exhibited higher survival following irradiation compared with parental controls, with no significant difference between P5 and P20 cells. All experiments were performed within 20 passages after cessation of irradiation.

### 4.16 Irradiation Procedure and Comet Assay

For DNA damage experiments, cells were irradiated using the same X‐ray system at a dose rate of approximately 1 Gy/min. Unless otherwise specified, a single dose of 4 Gy was delivered. DNA damage response was assessed by γ‐H2AX immunofluorescence staining and Western blot analysis. For immunofluorescence quantification, at least 30 cells per condition were analyzed in each independent experiment.

DNA strand breaks were further quantified using an alkaline comet assay. Following treatment, cells were embedded in low‐melting‐point agarose on comet slides and lysed in cold lysis buffer for at least 1 h. Slides were incubated in alkaline electrophoresis buffer for 20 min to allow DNA unwinding and subjected to electrophoresis at 25 V (0.8 V/cm) for 30 min at 4°C. After neutralization in 0.4 M Tris (pH 7.5), DNA was stained with propidium iodide. Comets were visualized under a fluorescence microscope, and tail moments (defined as the product of tail length and the fraction of DNA in the tail) were quantified using ImageJ with the OpenComet plugin. At least 50 cells per sample were analyzed.

### 4.17 Patient‐Derived Organoid (PDO) Culture

Three patient‐derived organoid (PDO) lines established from PanNETs, generated as previously described, displayed robust neuroendocrine differentiation with islet‐like morphology and positive immunostaining for synaptophysin (SYN) and chromogranin A (CGA) [12]. For organoid initiation, tumor specimens were minced into ∼1‐mm^3^ fragments and enzymatically digested in Advanced DMEM/F12 supplemented with 1.5 mg/mL collagenase II and 10 µM Y‐27632 (ROCK inhibitor) at 37°C for 1 h with gentle agitation. The resulting cell suspension was filtered (70‐µm strainer) and washed with cold PBS. Pelleted cells were resuspended in ice‐cold Matrigel and plated as drops in 24‐well plates (1–2 drops per well). After the Matrigel solidified (incubation at 37°C for 15 min), organoid cultures were maintained in two types of medium: a basal organoid medium (Advanced DMEM/F12 supplemented with HEPES, GlutaMAX, B27 supplement, N2 supplement, and Primocin) and an enriched organoid medium containing additional niche factors (10 µm Y‐27632, 1 µm A83‐01 TGF‐β inhibitor, 1 mm N‐acetylcysteine, 10 mm nicotinamide, 100 ng/mL FGF10, 50% Wnt3A‐conditioned medium, 10% R‐spondin1‐conditioned medium, 100 ng/mL Noggin, 10 µM forskolin). Organoids were cultured at 37°C with medium changes every 3–4 days. Growth was monitored by brightfield microscopy, and organoids were passaged using mechanical dissociation or trypsinization when they reached an appropriate size.

### 4.18 Animal Studies

All animal experiments were approved by the Institutional Animal Care and Use Committee (IACUC) of the Shanghai Experimental Animal Center (Approval No. FUSCC‐IACUC‐S2025‐0582). Female BALB/c nude mice (5 weeks old) were used for in vivo experiments. For the orthotopic PanNET xenograft model, 1 × 10^6^ luciferase‐expressing BON‐1 cells in 50 µL PBS/Matrigel (1:1 mix) were injected into the pancreatic tail of each mouse (*n* = 5 mice per group). Tumor growth in orthotopic models was monitored weekly by bioluminescence imaging (BLI). Mice were injected intraperitoneally with D‐luciferin (150 mg/kg) and imaged using the IVIS Spectrum system (PerkinElmer); signal intensity (photons/sec) was quantified to estimate tumor burden. For the patient‐derived xenograft (PDX) model, ∼2 mm^3^ fragments derived from PanNET patients (G1, G2, and G3) were surgically implanted subcutaneously into nude mice (one fragment per mouse, n = 5). Once established, PDX tumors were measured with calipers, and volumes were calculated as 0.5 × (length × width^2^). Tumor size measurements and volume calculations were performed by investigators blinded to treatment allocation during caliper‐based assessments. When xenograft tumors reached ∼1000 mm^3^, mice were humanely euthanized and the tumors were harvested. Small tumor fragments were then re‐implanted into new mice for expansion or further experiments as needed. Throughout all animal studies, mice were housed in specific pathogen‐free conditions with free access to food and water, and were closely observed for any signs of distress. Mice were randomly assigned to experimental groups for treatment or control conditions in intervention studies.

### 4.19 97491 Formulation, Dose Selection, and Plasma Exposure

The SIRT7 inhibitor 97491 was formulated in a 1:1 mixture of ethanol and Cremophor EL/95% as a 4× stock solution and diluted to the working concentration with sterile water immediately before use (oral gavage). To justify the in vivo dose used in efficacy studies, we performed a dose‐escalation tolerability pilot in five‐week‐old BALB/c nude mice. Mice received vehicle or escalating doses of 97491 (0.5, 1, 2.5, 5, 10, and 15 mg/kg) by oral gavage on a schedule matched to the efficacy experiment (once daily, 5 days/week for 2 weeks; 10 dosing days). Animals (*n* = 3 per dose) were monitored daily for clinical signs and body weight. The maximum tolerated dose (MTD) was predefined as the highest dose resulting in <15% body‐weight loss and no severe clinical toxicity or requirement for humane euthanasia. Doses at 10 mg/kg were at/near the MTD and were associated with lethargy and liver injury, whereas 15 mg/kg exceeded the MTD, resulting in ≥15% body‐weight loss and mortality. In contrast, 5 mg/kg was well tolerated with minimal body‐weight change and no overt toxicity, and was therefore selected as the working dose for the PDX efficacy studies (Table S12). Plasma exposure at each dose level was quantified by LC–MS/MS, and non‐compartmental pharmacokinetic parameters (Cmax, Tmax, AUC0–24, t1/2) are provided in Table S13.

### 4.20 Statistical Analysis

Unless otherwise specified, quantitative data are presented as mean ± standard error of the mean (SEM) from at least three independent experiments. Sample sizes (n) for each experiment are indicated in the corresponding figure legends or method descriptions. No data transformation was applied unless otherwise stated. Data normalization was performed as appropriate for each assay. No data points were excluded as outliers unless predefined criteria were specified. Comparisons between two groups were performed using unpaired two‐tailed Student's t‐tests, or paired two‐tailed Student's t‐tests for matched samples, as appropriate. Comparisons among multiple groups were performed using one‐way ANOVA. Correlation analyses were performed using Pearson correlation coefficients. Survival curves were analyzed using the log‐rank test. All statistical tests were two‐sided, and a *p* value < 0.05 was considered statistically significant. In figures, significance is indicated as ns, not significant; *p* < 0.05, *p* < 0.01, and *p* < 0.001. Statistical analyses were performed using GraphPad Prism version 9.5.1 and R.

## Author Contributions

J.Y.J. conceptualized the study, validated results, conducted the investigation, designed methodology, and drafted the manuscript. Y.W. contributed to methodology development and project management. Y.Q. obtained funding and coordinated the project. L.H.C. supplied resources and assisted with coordination. X.W.X. supervised the research and secured funding. G.X.F., D.S.J., and M.Y.W. contributed to methodology development. X.J.Y. oversaw the study and acquired funding. J.F.X. oversaw the project, supervised the work, and secured funding. S.R.J. conceived the study, supervised the project, and acquired funding. J. C. conceptualized the study, provided supervision, and acquired funding.

## Ethics Statement

All human and animal studies were conducted in compliance with institutional and national guidelines for research ethics. The Clinical Research Ethics Committee of Fudan University Shanghai Cancer Center approved the use of human tissues (Approval No. 2105235), and written informed consent was obtained from each patient. Animal protocols were approved by FUSCC‐IACUC (Approval No. FUSCC‐IACUC‐S2025‐0582), and all efforts were made to minimize animal suffering.

## Conflicts of Interest

The authors declare no conflicts of interest.

## Supporting information




**Supporting File 1**: advs75280‐sup‐0001‐SuppMat.docx.

**Supporting File 2**: advs75280‐sup‐0002‐Table S1‐S13.xlsx.

## Data Availability

The RNA‐seq raw data in this study have been deposited in the NCBI Gene Expression Omnibus (GEO) under accession number GSE323363. The ChIP‐PCR replicate data have been deposited in Mendeley Data (https://doi.org/10.17632/j2rjmxyyfw.1). Any additional information required to reanalyze the data reported in this work is available from the lead contact upon request. The data that support the findings of this study are available from the corresponding author upon reasonable request.
