# Supplementary material for: Inhibition of SIRT7 Overcomes Radioresistance in Pancreatic Neuroendocrine Tumors by Reactivating MEN1 Expression
Source: Adv Sci (Weinh). 2026 Apr 13;13(39):e19824. doi: 10.1002/advs.202519824 (PMC13335021; doi:10.1002/advs.202519824)
Supplement: Supplementary file 1 — Supporting File 1: advs75280‐sup‐0001‐SuppMat.docx. [file ADVS-13-e19824-s001.docx]

**Supplementary Figure legends**


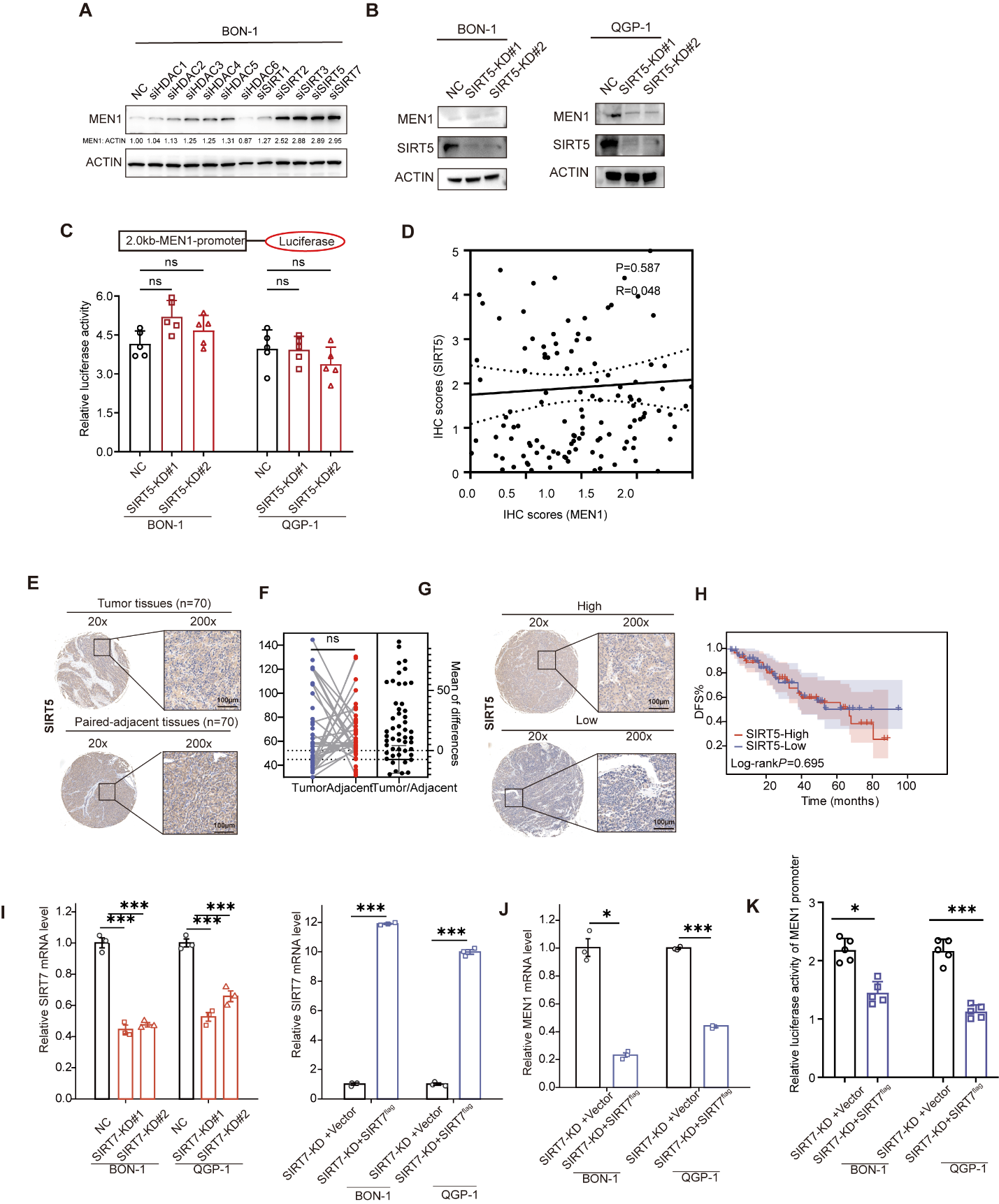


**Supplementary Figure 1. Validation of candidate sirtuins/HDACs from the epigenetic CRISPR screen and exclusion of SIRT5 as an upstream transcriptional regulator of MEN1.** (A) Representative immunoblot showing MEN1 protein levels following siRNA knockdown of HDAC and sirtuin family members in BON-1 cells (NC, negative control). n = 3 independent experiments. (B) Immunoblot validation of SIRT5 knockdown (SIRT5-KD#1 and SIRT5-KD#2) in BON-1 and QGP-1 cells. n = 3 independent experiments. (C) MEN1 promoter (−2.0 kb) luciferase reporter assay in BON-1 and QGP-1 cells following SIRT5 knockdown (SIRT5-KD#1 and SIRT5-KD#2) versus NC. n = 5 biologically independent samples per group. Statistics: one-way ANOVA. (D) Correlation analysis between SIRT5 and MEN1 IHC scores in a PanNET cohort (n = 121). Spearman correlation: R = 0.048, P = 0.587. (E) Representative IHC images of SIRT5 in paired tumor and adjacent tissues (n = 70 pairs). Images are shown at 20× and 200×; scale bar, 100 μm (200×). (F) Quantification of SIRT5 IHC in paired tumor versus adjacent tissues. Statistics: two-sided paired t-test. (G) Representative examples of SIRT5 IHC staining patterns in tumors with high versus low SIRT5 expression. Images are shown at 20× and 200×; scale bar, 100 μm (200×). (H) Kaplan–Meier analysis of disease-free survival (DFS) stratified by SIRT5-high versus SIRT5-low expression in the PanNET cohort (n = 121). Statistics: log-rank test, P = 0.695. (I) RT–qPCR confirmation of SIRT7 knockdown efficiency in BON-1 and QGP-1 cells. n = 3 biologically independent samples. Statistics: one-way ANOVA. (J) RT–qPCR analysis of SIRT7 mRNA following rescue (SIRT7-KD + SIRT7^Flag^) versus vector control (SIRT7-KD + Vector) in BON-1 and QGP-1 cells. n = 3 biologically independent samples. (K) MEN1 promoter luciferase reporter activity in BON-1 and QGP-1 cells after SIRT7 knockdown and rescue (SIRT7-KD + Vector vs SIRT7-KD + SIRT7^Flag^). n = 5 biologically independent samples per group. Statistics: unpaired two-sided Student’s t-test. Data are presented as mean ± SEM unless otherwise indicated. Significance is shown in the panels (ns, not significant; *P < 0.05, **P < 0.01, ***P < 0.001).


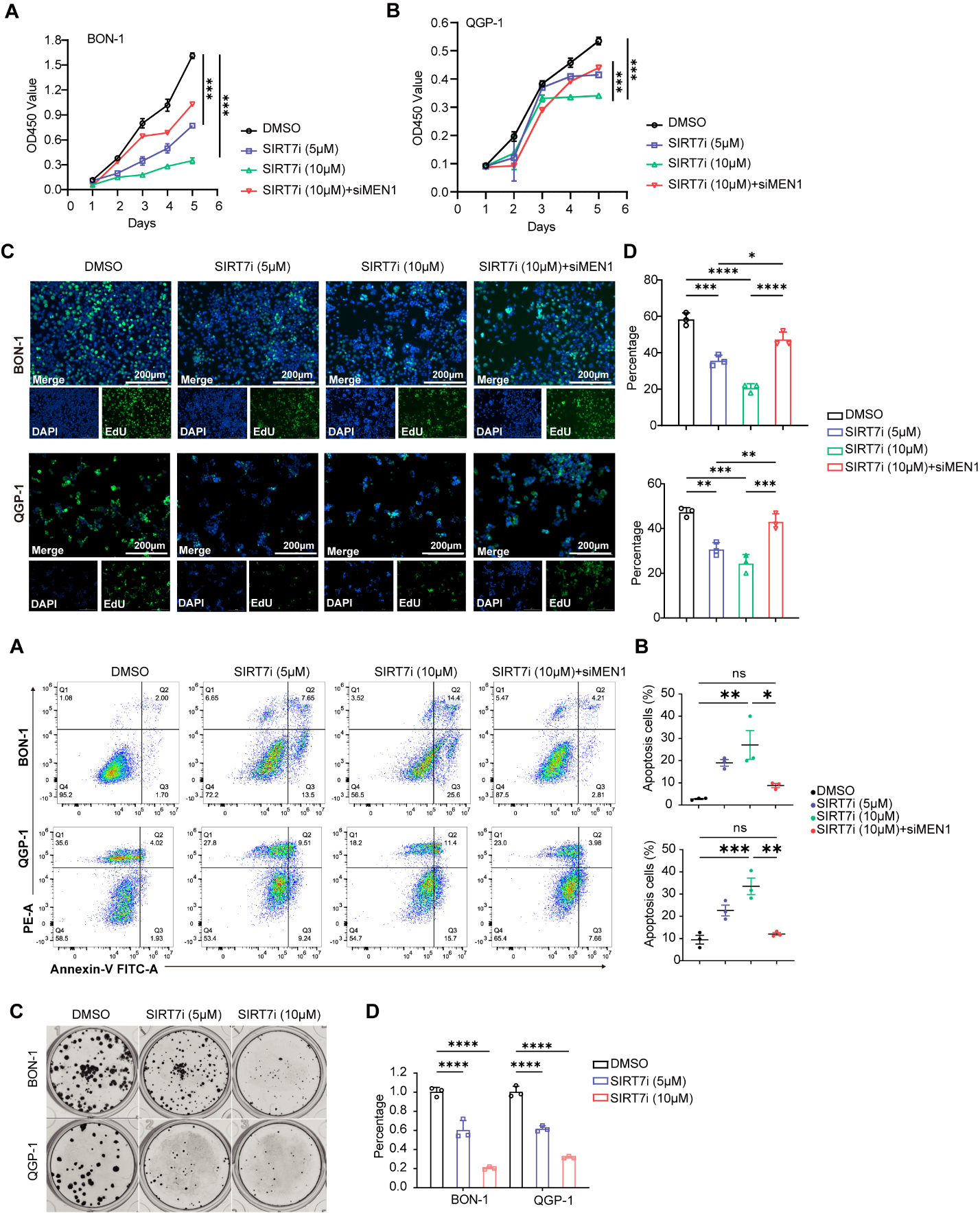


**Supplementary Figure S2. Pharmacological inhibition of SIRT7 recapitulates the anti-proliferative and pro-apoptotic effects observed with genetic SIRT7 depletion. (A)** Representative immunoblot showing MEN1 protein levels in BON-1 and QGP-1 cells under the indicated conditions; siMEN1 effectively reduced MEN1 protein levels in both control and SIRT7-KD cells. (B) Cell viability of BON-1 and QGP-1 cells treated with DMSO, SIRT7 inhibitor (SIRT7i; 5 μM or 10 μM), or SIRT7i (10 μM) combined with siMEN1 over a 6-day time course, measured by CCK-8 assay. n = 3 biologically independent experiments per group. Statistics: one-way ANOVA. (C) Representative EdU incorporation images in BON-1 and QGP-1 cells under the indicated treatments. Nuclei were counterstained with DAPI (blue), and proliferating cells were labeled with EdU (green). Scale bars, 200 μm. (D) Quantification of EdU-positive cells. n = 3 biologically independent experiments per group. Statistics: one-way ANOVA. (E) Representative flow cytometry plots of Annexin V-FITC/PI staining in BON-1 and QGP-1 cells following the indicated treatments. (F) Quantification of apoptotic cells (Annexin V-positive). n = 3 biologically independent experiments per group. Statistics: one-way ANOVA. (G) Representative colony formation assays of BON-1 and QGP-1 cells treated with DMSO or SIRT7i. (H) Quantification of clonogenic survival. n = 3 biologically independent experiments per group. Statistics: one-way ANOVA. Data are presented as mean ± SEM (ns, not significant; *P < 0.05, **P < 0.01, ***P < 0.001, ****P < 0.0001).


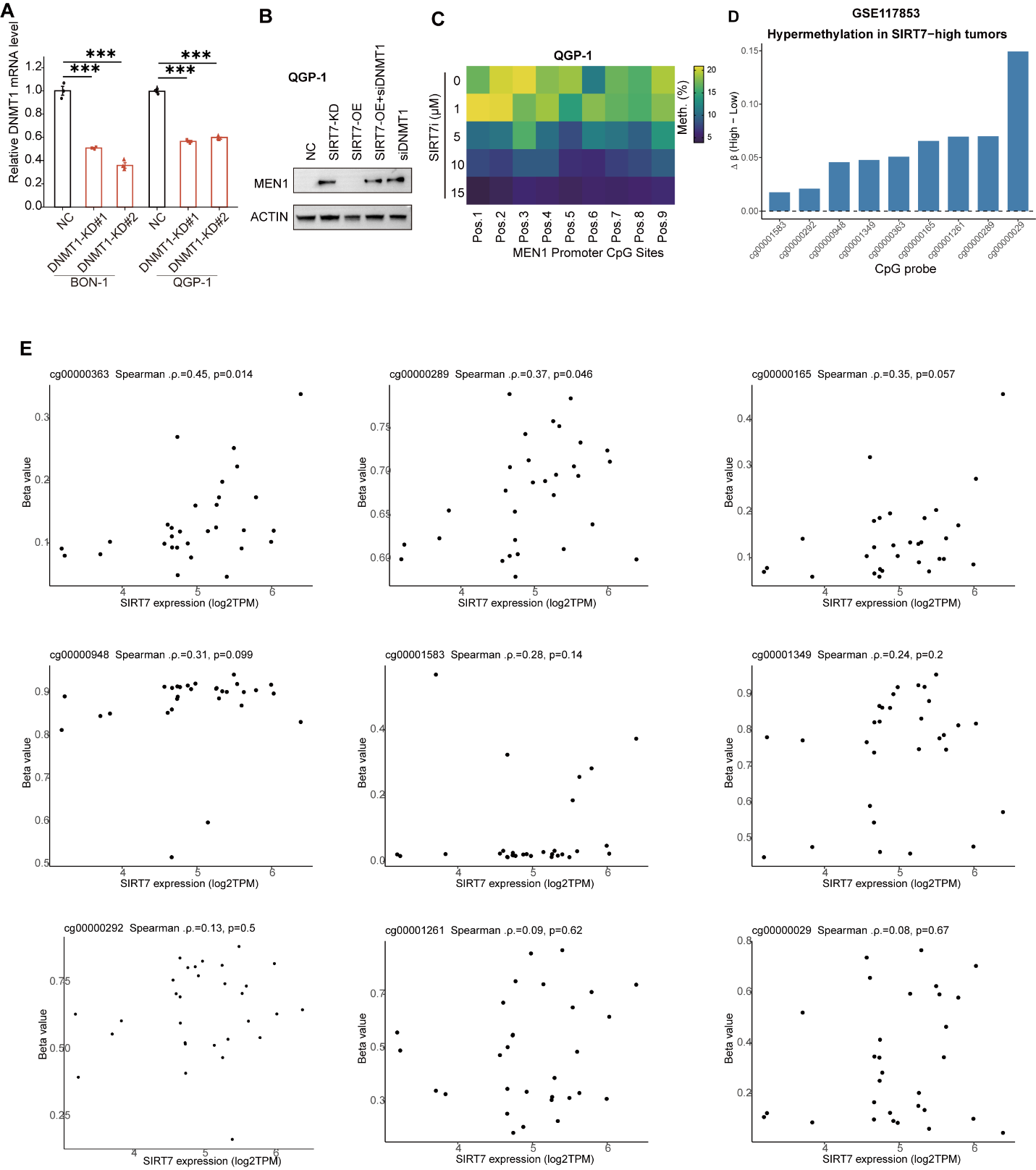


**Supplementary Figure 3. DNMT1 mediates SIRT7-dependent methylation and transcriptional repression of the MEN1 promoter in PanNET cells. (A)** RT–qPCR validation of DNMT1 knockdown efficiency in BON-1 and QGP-1 cells (DNMT1-KD#1 and DNMT1-KD#2). n = 3 biologically independent experiments. Statistics: one-way ANOVA. **(B)** Representative immunoblot of MEN1 protein levels in QGP-1 cells under the indicated conditions. n = 3 independent experiments. **(C)** Quantitative pyrosequencing heatmap showing methylation percentages of nine CpG sites (Pos.1–Pos.9) within the MEN1 promoter in QGP-1 cells treated with increasing concentrations of SIRT7 inhibitor (SIRT7i; 0–15 μM). Data represent mean methylation levels from three independent experiments. **(D)** Analysis of a public PanNET cohort with matched Illumina 450K DNA methylation and RNA expression data (GSE117853, n = 30). Nine CpG probes mapping within or immediately adjacent to the same promoter-proximal CpG island interrogated by our pyrosequencing amplicon were identified. Tumors were stratified by SIRT7 expression (top vs bottom tertile, n = 10 per group). **(E)** Spearman correlation analysis between SIRT7 expression (log2TPM) and methylation β values of individual CpG probes in GSE117853 (n = 30). Correlation was assessed by two-sided Spearman rank test.


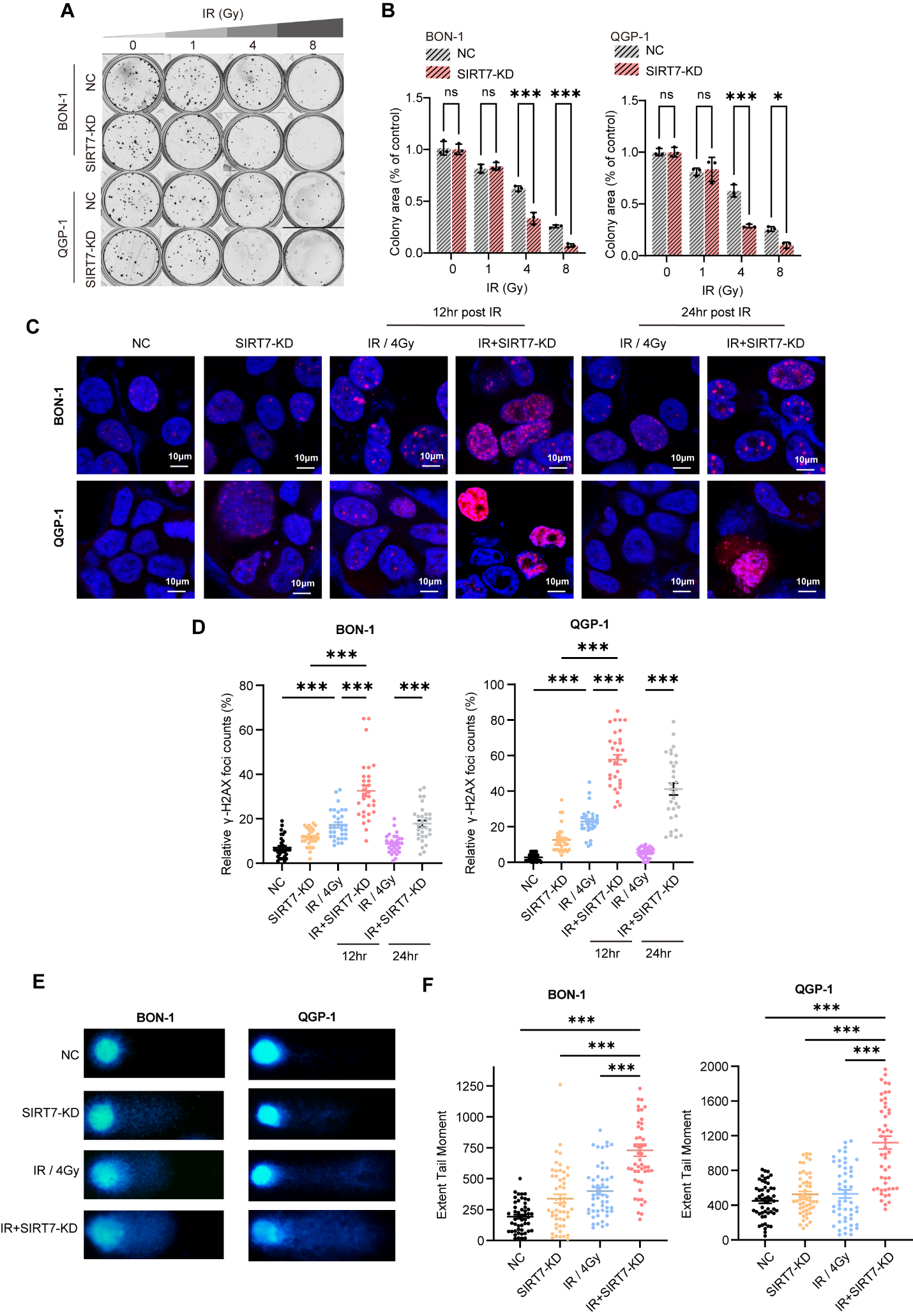


**Supplementary Figure 4. Genetic depletion of SIRT7 sensitizes PanNET cells to irradiation and impairs DNA double-strand break repair. (A)** Representative colony-formation images of BON-1 and QGP-1 cells transduced with control shRNA (NC) or SIRT7 shRNA (SIRT7-KD) and exposed to increasing doses of ionizing radiation (IR; 0, 1, 4, and 8 Gy). **(B)** Quantification of colony area (percentage of non-irradiated control) corresponding to (A). n = 3 biologically independent experiments. Statistics: two-sided unpaired Student’s t-test. **(C)** Representative immunofluorescence images of γ-H2AX foci in BON-1 and QGP-1 cells under the indicated conditions at 12 h and 24 h post-irradiation. Nuclei were counterstained with DAPI. Scale bar, 10 μm. **(D)** Quantification of γ-H2AX foci counts per nucleus corresponding to (C). n = 3 biologically independent experiments. Statistics: one-way ANOVA. **(E, F)** Representative comet assay images and quantification of tail moment in BON-1 and QGP-1 cells at 12 h post-treatment under indicated conditions. n = 3 biologically independent experiments. Statistics: one-way ANOVA. Data are presented as mean ± SEM. (ns, not significant; *P < 0.05, **P < 0.01, ***P < 0.001, ****P < 0.0001).


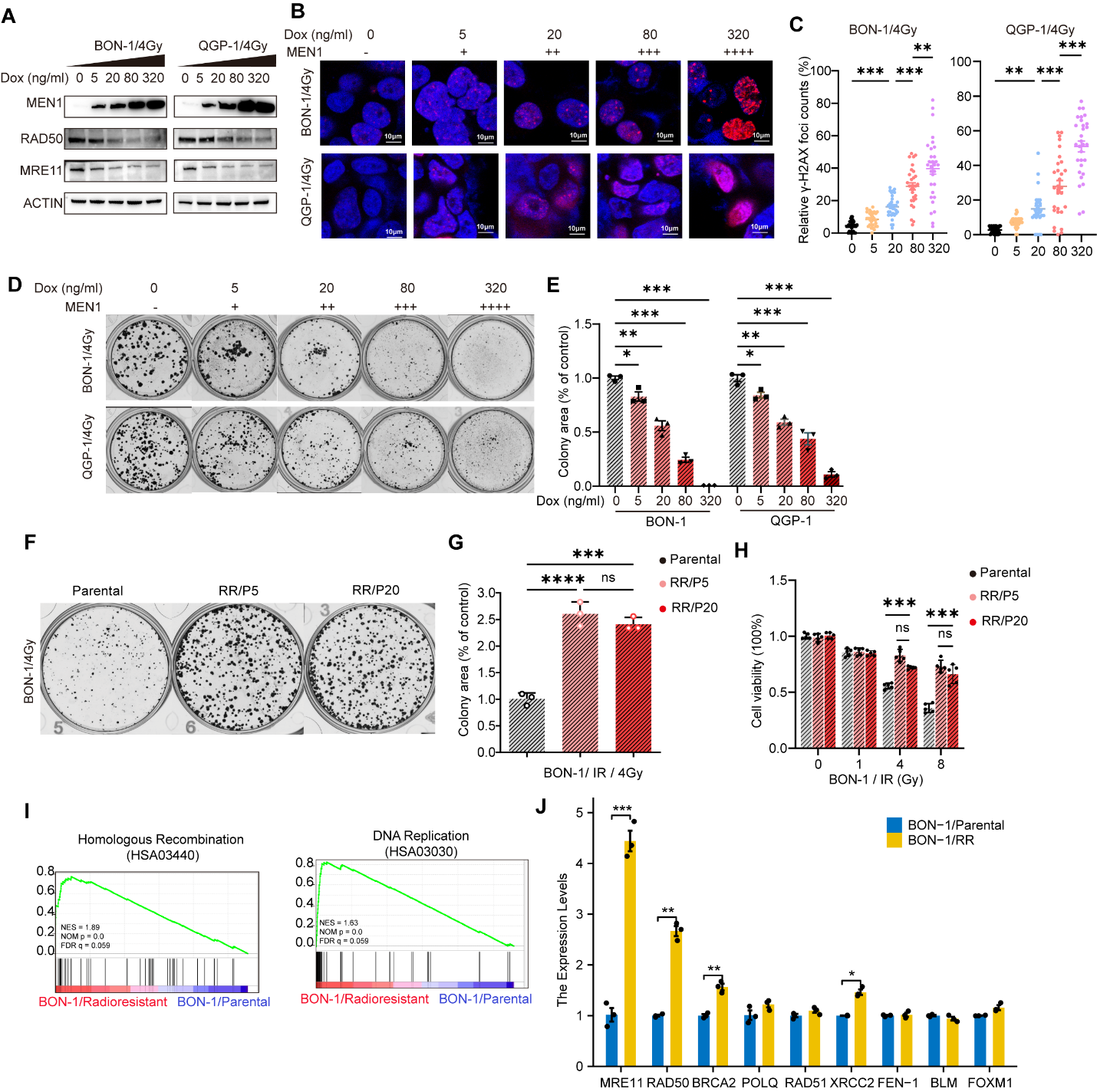


**Supplementary Figure 5. Establishment and characterization of a stable radioresistant BON-1 model and graded MEN1 re-expression analysis.** (A) Western blot analysis of MEN1, RAD50, and MRE11 protein levels in MEN1-KO BON-1 and QGP-1 cells carrying a doxycycline-inducible MEN1 re-expression system. Cells were treated with increasing doxycycline concentrations and harvested 12 h after 4 Gy irradiation. n = 3 biologically independent experiments. (B) Representative immunofluorescence images of γ-H2AX foci formation in BON-1 and QGP-1 cells under graded MEN1 re-expression at 12 h after 4 Gy irradiation. Nuclei were counterstained with DAPI. Scale bars, 10 μm. (C) Quantification of γ-H2AX foci corresponding to (B). n = 3 biologically independent experiments. Statistics: one-way ANOVA. (D) Representative colony formation images of BON-1 and QGP-1 cells under graded MEN1 re-expression after 4 Gy irradiation. (E) Quantification of colony area (% of control) corresponding to (D). n = 3 biologically independent experiments. Statistics: one-way ANOVA. (F) Representative colony formation images of parental BON-1 cells and radioresistant sublines (RR/P5 and RR/P20) after 4 Gy irradiation. (G) Quantification of colony area corresponding to (F). n = 3 biologically independent experiments. Statistics: one-way ANOVA. (H) Cell viability of parental BON-1, RR/P5, and RR/P20 cells following graded irradiation. n = 5 biologically independent experiments. Statistics: one-way ANOVA. (I) GSEA comparing parental and radioresistant BON-1 cells, showing enrichment of Homologous Recombination (HSA03440; NES = 1.89, NOM p = 0.0, FDR q = 0.059) and DNA Replication (HSA03030; NES = 1.63, NOM p = 0.0, FDR q = 0.059) pathways in resistant cells. (J) RT–qPCR validation of DDR-related gene expression (MRE11, RAD50, BRCA2, POLQ, RAD51, XRCC2, FEN1, BLM, FOXM1) in parental versus BON-1/RR cells. n = 3 biologically independent experiments. Statistics: unpaired two-sided Student’s t-test. Data are presented as mean ± SEM unless otherwise indicated (ns, not significant; *P < 0.05; **P < 0.01; ***P < 0.001; ****P < 0.0001).


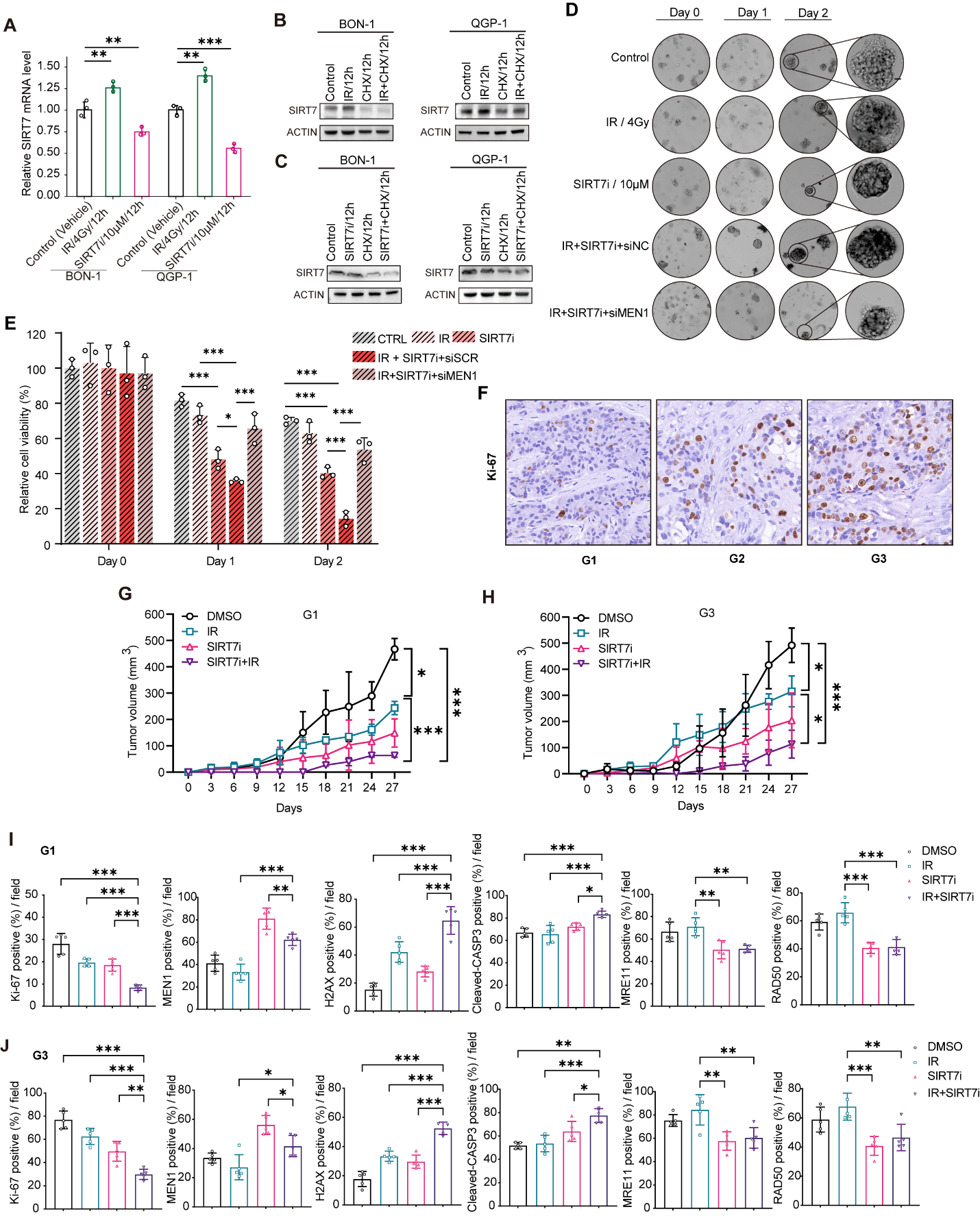


**Supplementary Figure 6. Transcriptional regulation of SIRT7 by irradiation and validation of combination treatment effects in additional PDX models.** (A) RT–qPCR analysis of SIRT7 mRNA expression in BON-1 and QGP-1 cells treated with vehicle (Control), ionizing radiation (IR, 4 Gy), or SIRT7 inhibitor (97491, 10 μM) for 12 h. n = 3 independent experiments. (B, C) Cycloheximide (CHX) chase assays evaluating SIRT7 protein stability in BON-1 and QGP-1 cells. Cells were treated with IR (4 Gy) or 97491 (10 μM) for 12 h, in the presence or absence of CHX (10 μg/mL). n = 3 independent experiments. (D, E) Patient-derived PanNET organoids (PDOs) treated with SIRT7i (10 μM) and/or IR (4 Gy) and monitored over time. (D) Representative bright-field images at the indicated days. (E) Quantification of organoid viability (relative to day 0). n = 3 independent PDO cultures (biological replicates). Statistics: one-way ANOVA. (F) primary tumors of patients at different grades (G1, G2, G3). (G, H) Tumor growth curves of additional G1 (G) and G3 (H) PanNET PDX models treated with DMSO (vehicle), IR (2 Gy/fraction, 3 days/week), SIRT7 inhibitor (5 mg/kg/day), or combination therapy. Tumor volumes were measured every 3 days. n = 5 mice per group. (I, J) Quantification of IHC staining in G1 (I) and G3 (J) PDX tumors at endpoint, including Ki-67 (proliferation), MEN1, γ-H2AX (DNA damage), cleaved CASP3 (apoptosis), MRE11, and RAD50. Statistics: one-way ANOVA. Data are presented as mean ± SEM unless otherwise indicated (ns, not significant; *P < 0.05; **P < 0.01; ***P < 0.001; ****P < 0.0001).


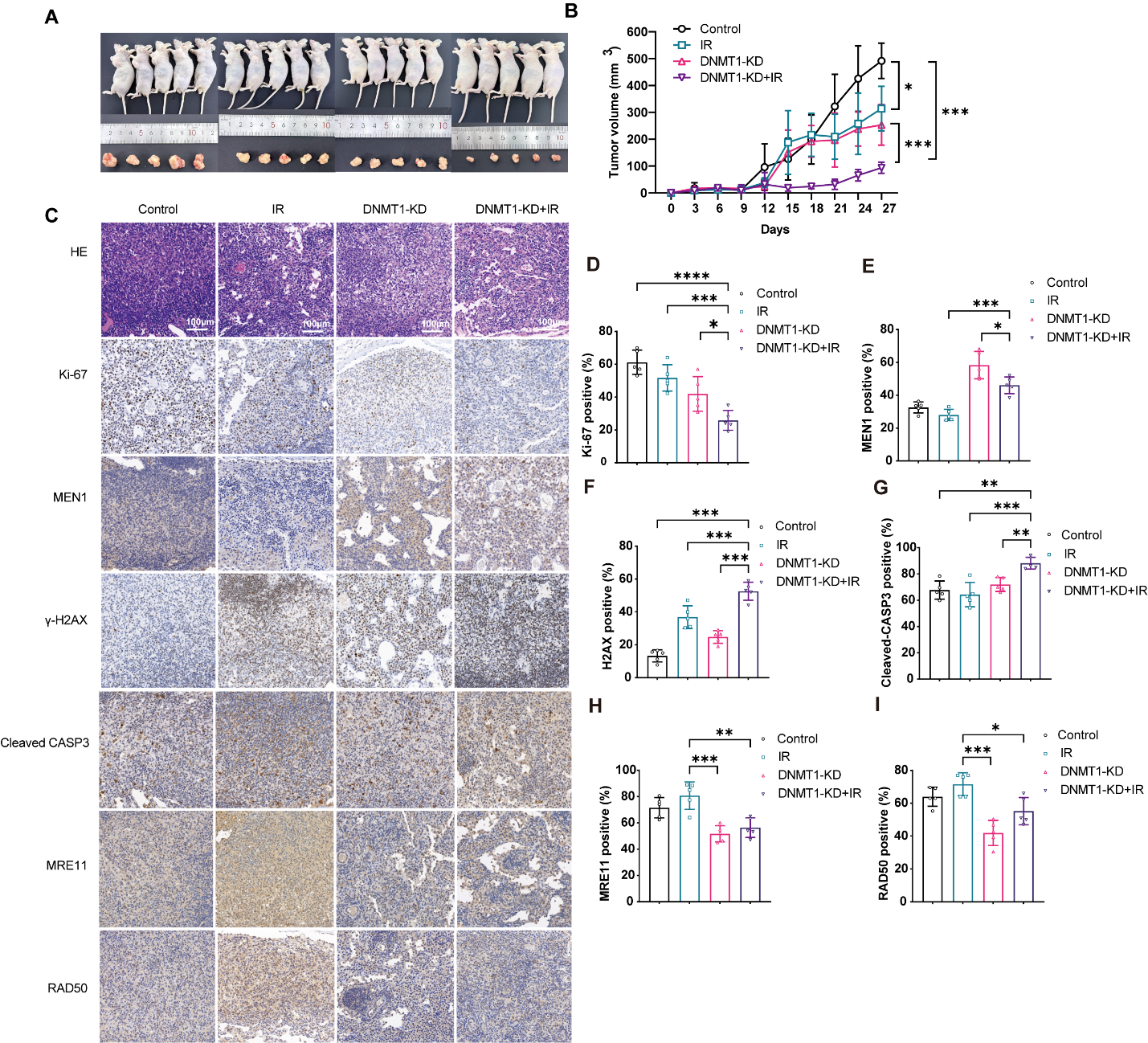


**Supplementary Figure 7. DNMT1 knockdown enhances irradiation response in vivo in PanNET xenograft models.** **(A)** Representative images of mice and excised subcutaneous tumors from the four treatment groups at endpoint in BON-1–derived xenograft models. **(B)** Tumor growth curves over time in mice bearing BON-1 control or DNMT1-KD xenografts with or without irradiation. Tumor volumes were measured every 3 days. n = 5 mice per group. Statistical comparisons at endpoint were performed using one-way ANOVA. **(C)** Representative hematoxylin and eosin (H&E) and immunohistochemistry (IHC) staining of tumor sections for Ki-67, MEN1, γ-H2AX, Cleaved CASP3, MRE11, and RAD50 across the four treatment groups. Images were acquired at 200× magnification. Scale bar, 100 μm. **(D–I)** Quantification of IHC staining: (D) Ki-67-positive cells (% per field), (E) MEN1-positive cells (% per field), (F) γ-H2AX-positive cells (% per field), (G) Cleaved CASP3-positive cells (% per field), (H) MRE11-positive cells (% per field), (I) RAD50-positive cells (% per field). n = 5 tumors per group. Statistical analysis was performed using one-way ANOVA. Data are presented as mean ± SEM (ns, not significant; *P < 0.05; **P < 0.01; ***P < 0.001; ****P < 0.0001).
